# Multi-omics analysis reveals the efficacy of two probiotic strains in managing feline chronic kidney disease through gut microbiome and host metabolome

**DOI:** 10.3389/fvets.2025.1590388

**Published:** 2025-06-18

**Authors:** Hsiao-Wen Huang, Tzu-Chien Kuo, Ya-Jane Lee, Ming-Ju Chen

**Affiliations:** ^1^Department of Animal Science and Technology, National Taiwan University, Taipei, Taiwan; ^2^Institute of Veterinary Clinical Science, School of Veterinary Medicine, National Taiwan University, Taipei, Taiwan; ^3^Department of Internal Medicine, National Taiwan University Veterinary Hospital, Taipei, Taiwan; ^4^Center for Biotechnology, National Taiwan University, Taipei, Taiwan

**Keywords:** chronic kidney disease, colonization efficiency, gut dysbiosis, gut microbiome, host metabolome, host-microbe interactions, *Lactobacillus* mix

## Abstract

Gut dysbiosis has been implicated in the progression of chronic kidney disease (CKD), yet the functional alterations of the microbiome and their links to host metabolism in feline CKD pathophysiology remain unclear. Our previous findings suggested that *Lactobacillus* mix (Lm) may mitigate CKD progression by modulating gut microbiota composition and restoring microbial balance. In this pilot study, we aimed to evaluate the potential effects of an 8-week Lm intervention in cats with stage 2–3 CKD and to investigate the underlying host-microbiota interactions through integrated multi-omics analysis. We performed full-length 16S rRNA amplicon sequencing and untargeted metabolomics to characterize the intricate interactions between the gut microbiome and host metabolome, and further investigate the modulation of microbial function and its related gut-derived metabolites before and after the intervention. During this period, creatinine and blood urea nitrogen levels were stabilized or reduced in most cats, and gut-derived uremic toxins (GDUTs) showed modest numerical reductions without statistically significant changes. Lm intervention was also associated with increased gut microbial diversity, alterations in specific bacterial taxa, and upregulation of microbial functions involved in GDUTs and short-chain fatty acid (SCFAs) biosynthesis pathways. To further explore individual variations in response, we conducted a *post hoc* exploratory subgroup analysis based on changes in microbial-derived metabolites. Cats classified as high responders, defined as those with reductions in three GDUTs and increases in SCFAs, exhibited distinct microbiome compositions, microbial functional profiles, and metabolite shifts compared to moderate responders. Among high responders, modulation of microbial pathways involved in GDUTs (tyrosine, tryptophan, and phenylalanine metabolism) and SCFAs (pyruvate, propanoate, and butanoate metabolism) biosynthesis was particularly evident. Notably, the relative abundance of Lm strains was higher in high responders, suggesting a potential association between colonization efficiency and microbial metabolic outcomes. This study demonstrates an Lm-mediated interconnection between the modulation of microbial composition, metabolic functions, and systemic metabolite profiles. Overall, our findings suggest that Lm intervention may influence the gut-kidney axis in cats with CKD. These preliminary, hypothesis-generating results highlight the value of multi-omics approaches for understanding host-microbe interactions and support further investigation into personalized probiotic strategies as potential adjuvant therapies in feline CKD.

## 1 Introduction

Chronic kidney disease (CKD) is one of the most common diseases in humans, as well as in elderly cats. It is characterized by a decline in kidney function and the accumulation of harmful uremic retention toxins in the circulation (1, 2). Indoxyl sulfate (IS) and *p*-cresyl sulfate (PCS), which are primarily classified as protein-bound uremic toxins, are gut microbial-derived metabolites that exist in both free and bound forms in circulation (3, 4). Both toxins have detrimental effects on renal, vascular, and cardiac tissues (5). Thus, increased circulating levels of IS and PCS are negatively correlated with kidney function in both humans, dogs and cats, and are strongly associated with a decline in the estimated glomerular filtration rate (4, 6–8).

The gut microbiota is vital for maintaining host health and affects several pathological conditions associated with gut dysbiosis (9, 10). Therefore, disturbance in normal gut microbiota can accelerate CKD progression (11, 12). CKD drives the transition from a more even and diverse microbial community to a more dominant and identical composition in the intestinal environment by reducing the number of saccharolytic bacterial species and increasing proteolytic ones (13, 14). This leads to the generation of uremic metabolites and aggravates kidney failure (15–18). Without the effective amelioration of gut dysbiosis, kidney function may persistently deteriorate and accelerate disease progression.

Microbiota-based therapeutic approaches increase the number of symbiotic and beneficial bacteria to harmonize the microbial composition and restore gut homeostasis (19). Probiotics are widely recognized for modulating gut microbiota and positively impacting the pathophysiology of various diseases through the gut-organ axis (20, 21). They are considered practical adjuvant therapies for reducing uremic toxins and further slowing the underlying CKD progression. In our previous study, we explored two strains, *Lactiplantibacillus plantarum* subsp. *plantarum* MFM 30-3 and *Lacticaseibacillus paracasei* subsp. *paracasei* MFM 18, isolated from Mongolian fermented milk and noted for their ability to clear precursors of uremic toxins, collectively named *Lactobacillus* mix (Lm). Our findings revealed that Lm alleviated adenine-induced CKD by improving gut dysbiosis and restoring the abundance of commensal bacteria in the gut, especially short-chain fatty acids (SCFAs) producers. This led to the reduction of uremic toxins and the prevention of intestinal barrier disruption via the modulation of microbial composition and metabolite production (22).

Numerous multi-omics studies in humans and rodent models have provided conclusive evidence that gut dysbiosis contributes to the progression of chronic kidney disease (CKD) by altering gut-derived metabolites and aggravating kidney dysfunction (13, 14, 23, 24). Recent studies in cats with CKD have demonstrated similar patterns, including reduced fecal bacterial diversity and richness, along with elevated levels of gut-derived uremic toxins (GDUTs) such as IS (7). Despite these findings, comprehensive multi-omics investigations into the roles of the gut microbiome and metabolome in feline CKD remain limited. Therefore, we hypothesized that multi-omic analyses could offer new insights into the potential of Lm supplementation by elucidating host-microbe interactions and their relevance to CKD pathophysiology in cats. In this study, we conducted an open-label, single-arm pilot study to investigate the effect potential of an 8-week Lm intervention in cats with stage 2–3 CKD. To gain insights into the mechanisms underlying host-microbe interactions, we performed multi-omics analyses of the gut microbiome and serum metabolome before and after the intervention.

While assessing overall changes in microbial composition and associated metabolites, we also observed notable inter-individual variability in biological responses. To further investigate this variation, we performed a *post hoc* exploratory subgroup analysis based on changes in microbial derived metabolites, specifically gut-derived uremic toxins (GDUTs) and short-chain fatty acids (SCFAs). This approach was intended to generate hypotheses about potential associations between microbial metabolite shifts, microbial function, colonization patterns, and host responses. By stratifying cats based on metabolite response profiles, we aimed to examine whether distinct microbiome and metabolome signatures could help explain differential outcomes and uncover potential precision mechanisms that govern probiotic efficacy. This perspective offers novel insights into the microbiome- and host metabolome-associated mechanisms that may influence the variability in probiotic efficacy for managing feline CKD.

## 2 Materials and methods

### 2.1 Study design

This single-arm pilot study was conducted at National Taiwan University Veterinary Hospital in Taiwan, from January to August 2021. The study was approved by the Institutional Animal Care and Use Committee of National Taiwan University (IACUC; Approval No. NTU-109-EL-00127) and Clinical Trial/Research Approval of National Taiwan University Veterinary Hospital (NTUVH; Approval No. 000081). All owners signed an informed consent form before allowing their cats to participate in the study.

### 2.2 Recruitment of participants

The inclusion criteria were based on the International Renal Interest Society criteria (25). This study recruited cats with stage 2–3 CKD with 1.6–5.0 mg/dL serum creatinine (CRE) or 18–38 μg/dL symmetric dimethylarginine (SDMA), both indicators of kidney function. In addition, participating cats showed one of the following conditions: a history of clinical signs (e.g., polyuria and polydipsia), abnormal urinalysis (urine specific gravity <1.035 or persistent proteinuria based on urine protein-to-creatinine ratio (UPC) >0.4 on three occasions 2 weeks apart) for at least 3 months. There were no limitations on age, sex, weight, breed, and sterilization. Cats with CKD were excluded from the study if they had acute kidney disease or acute worsening azotemia (>0.3 mg/dL increase in creatinine (CRE) concentration within 7 days), concurrent hyperthyroidism, lower urinary tract disease, diabetes, or clinical signs of other nonrenal diseases (hepatic, cardiac, neoplastic disease, gastrointestinal, or infection) in their medical records. In addition, cats with CKD were excluded if they had received antibiotics or probiotic-containing products within 4 weeks prior to the start of the study.

### 2.3 *Lactobacillus* mix intervention

To evaluate the clinical application of *Lactobacillus* mix (Lm), consisting of *Lactiplantibacillus plantarum* subsp. *plantarum* MFM 30-3 and *Lacticaseibacillus paracasei* subsp. *paracasei* MFM 18, cats with CKD were fed one probiotic capsule by their owner daily for 8 weeks. Each probiotic capsule contained a total of 5 × 10^9^ CFU of Lm, consisting of equal proportions of each strain (2.5 × 10^9^ CFU per strain). The selected dosage was based on a previously effective dose in a CKD mouse model (22) and was scaled for feline using established interspecies dose conversion methods (26). The cats maintained regular CKD therapy and their original dietary habits at the time of enrollment and during the study period.

### 2.4 Sample collection

First-morning urine, fasting blood, and fecal samples were collected from cats with CKD at 0 (baseline), 4, and 8 weeks following Lm intervention for clinical and multi-omics analyses. Fasting was defined as no food intake for at least 8 h, with compliance confirmed by owner reports and veterinary staff at the time of sampling. Urine samples were collected by owners at home using a clean plastic barrier placed over the litter, transferred into sterile tubes, stored at 4°C, and transported to the laboratory within 2 h using a portable cooler with ice packs. Blood samples were centrifuged at 1,000 × *g* for 10 min, and plasma was aliquoted and stored at −80°C for analyses of creatinine (CRE), blood urea nitrogen (BUN), symmetric dimethylarginine (SDMA), gut-derived uremic toxins (GDUTs), electrolyte concentrations, and metabolomics. Anticoagulated blood and urine were used for complete blood count and urinalysis, respectively. Fecal samples were collected by owners immediately after defecation using sterile tubes without preservative buffers, stored at −18°C at home, and transported to the laboratory under cooled conditions within 2 h. Upon arrival, samples were stored at −80°C until further processing for short-chain fatty acids (SCFAs) and gut microbiome analyses. Only the baseline (0-week) and 8-week plasma and fecal samples were subjected to multi-omics analyses (microbiome and metabolome profiling).

### 2.5 Gut-derived uremic toxins analysis

The concentrations of gut-derived uremic toxins (GDUTs) in plasma, such as trimethylamine-N-oxide (TMAO), indoxyl sulfate (IS), *p*-cresyl sulfate (PCS), and phenyl sulfate (PS), were measured as follows: Fifty microliters of plasma were added to 50 μL of isotopically labeled internal standards (1,000 ng/mL of PCS-d7, IS-d4, and PS-^13^C_6_ and 100 ng/mL of TMAO-d9) and 400 μL acetonitrile (ACN). The plasma mixtures were subsequently sonicated for 10 min. The samples were centrifuged at 16,100 × *g* for 15 min at 4°C, and 200 μL of the obtained supernatants was vacuum-concentrated and dissolved in 200 μL of 20% ACN for liquid chromatography-tandem mass spectrometry analysis. Gut-derived uremic toxins (GDUTs) and their corresponding internal standards were analyzed using the AB Sciex ExionLC AC high-performance liquid chromatography (HPLC) system coupled with an AB Sciex 5500 TripleQuad mass spectrometer (SCIEX, Framingham, MA, United States). Next, separation was performed using an Acquity UPLC BEH C18 Column (2.1 × 150 mm, 1.7 μm; Waters Corporation, Milford, MA, United States). The mobile phase comprised solvent A (0.1% formic acid in water) and solvent B (10 mM ammonium acetate in ACN). The injection volume was 5 μL, and the flow rate was 0.3 mL/min with a linear gradient elution over 6 min. The eluting gradient was as follows: 0.0–3.0 min (10–95% solvent B), 3.0–4.0 min (95% solvent B), 4.0–4.1 min (95–10% solvent B), and 4.1–6.0 min (0% solvent B). The positive electrospray ionization mode was set at the following parameters: 55 psi for nebulizer pressure, 55 psi for drying gas pressure, 5.5 kV for capillary voltage, and 550°C for drying gas temperature. The negative electrospray ionization mode was set at the same parameters but with a −4.5 kV capillary voltage. The mass spectrometer was configured in multiple reaction monitoring modes, and the monitored transition for TMAO was *m*/*z* 76 → 58 and 76 → 59; TMAO-d9 was *m*/*z* 85 → 66; IS was *m*/*z* 212 → 80 and 212 → 132; IS-d4 was *m*/*z* 216 → 80; PCS was *m*/*z* 187 → 107 and 187 → 80; PCS-d7 was *m*/*z* 194 → 114; PS was *m*/*z* 173 → 93 and 173 → 80; and PS-^13^C_6_ was *m*/*z* 179 → 99. The concentration of each uremic toxin in the samples was calculated based on calibration curves using the peak area ratio of the uremic toxin to its corresponding isotope internal standard.

### 2.6 Short-chain fatty acid measurement

The concentrations of fecal short-chain fatty acids (SCFAs), including acetate, propionate, and butyrate, were measured as previously described with slight modifications (27). Samples were homogenized in 70% ethanol for 45 s (FastPrep-24^™^ 5G Instrument, MP Biomedicals, Irvine, CA, United States) and centrifuged at 16,100 × *g* for 10 min at 4°C. The supernatants were then collected as the analytical samples. After a series of procedures, the obtained fatty acids were dissolved in 200 μL methanol. The concentration of SCFAs were measured using HPLC with a Reprosil 100 C18 column (250 × 4.6 mm, 5 μm particle size; Dr. Maisch GmbH, Ammerbuch-Entringen, Germany). The mobile phase consisted of ACN, methanol, and ultrapure water (30:16:54), and the pH was adjusted to 4.5 using 0.1% trifluoroacetic acid. The injection volume was 30 μL, the flow rate was 1.1 mL/min, the column oven temperature was 50°C, and the wavelength was 400 nm.

### 2.7 Microbiome analysis

Genomic DNA was extracted from fecal samples following a previously described protocol (28) with minor modifications. Briefly, fecal material was homogenized (FastPrep-24^™^ 5G Instrument) in extraction buffer with bead beating, followed by phenol-chloroform extraction and isopropanol precipitation. DNA was resuspended in TE buffer and stored at −20°C. The DNA concentration was measured using the Qubit 4.0 fluorometer (Thermo Fisher Scientific, Waltham, MA, United States) and adjusted to 1 ng/μL for subsequent experiments. The SMRTbell library was prepared by amplifying the full-length 16S rRNA genes (V1–V9 regions) using barcoded 16S rRNA gene-specific primers (forward: 5′-AGRGTTYGATYMTGGCTCAG-3′/reverse: 5′-RGYTACCTTGTTACGACTT-3′). Genes were sequenced using the PacBio Sequel IIe instrument (Menlo Park, CA, United States) in circular consensus sequence (CCS) mode to generate HiFi reads with a predicted accuracy (Phred Scale) of 30. Raw sequence data were de-multiplexed, and low-quality reads were filtered using the following quality thresholds: maximum expected errors (maxEE) = 2, minimum quality score (minQ) = 3, read length between 1,240–1,540 bp, and no ambiguous bases (maxN = 0). The CCS data were processed using DADA2 (dada2_1.20), which performs dereplication, error model learning, ASV inference, and chimera removal. DADA2 resolves exact amplicon sequence variants (ASVs) with single-nucleotide resolution from the full-length 16S rRNA gene with a near-zero error rate by accurately identifying and correcting nearly all sequencing errors, thereby yielding highly precise ASVs with minimal artifacts (29, 30). Representative sequences were processed and analyzed using QIIME2 (v2021.4), and the annotated taxonomy classification was assigned using the NCBI database (retrieved 2020.7) (31–36). Similarly, alpha diversity indices (observed species, Shannon, and Margalef’s richness) were analyzed using QIIME2. The observed features provide microbial richness based on ASV-identified counts. Shannon index consider both the richness and evenness. Margalef’s richness index is used to measure species richness (37–39). The rarefaction curve was constructed using R v4.0.3 with the phyloseq package. Dissimilarities among microbial communities were measured through Bray-Curtis distance and principal coordinates analysis (PCoA) to determine beta diversity (40, 41). PCoA was conducted using the phyloseq, vegan3d, and ggplot2 packages in R v4.0.3. Rarefaction was not applied prior to alpha or beta diversity analyses in order to retain full sequencing depth for downstream comparisons. Differences in sequencing depth between samples may introduce bias in diversity estimates and were considered during interpretation. Functional abundances from 16S rRNA sequencing data were analyzed to predict functional genes using the Tax4Fun2 package with the Ref99NR reference dataset (42). Abundances of the Kyoto Encyclopedia of Genes and Genomes (KEGG) orthology and pathways were normalized based on the total reads of individual cats with CKD for downstream analysis.

### 2.8 Untargeted metabolomic analysis

Fifty microliters of plasma sample were mixed with 150 μL methanol, vortexed for 30 s, sonicated for 10 min in an ice-water bath, and incubated for 1 h at −20°C to precipitate proteins. Next, the sample was centrifuged at 13,400 × *g* for 15 min at 4°C, and the supernatant was collected in a glass vial for further analysis. An equal volume of supernatants from each sample was mixed and utilized as quality control. Mass spectrometry was performed on a vanquish-focused ultra-high-performance liquid chromatography system coupled with a high-resolution Orbitrap Elite mass spectrometer (UHPLC-HRMS; Thermo Fisher Scientific) using electrospray ionization. Separation was performed using an Acquity BEH C18 Column (2.1 × 100 mm, 1.7 μm; Waters Corporation). The mobile phase comprised solvent A (0.1% formic acid in water) and solvent B (0.1% formic acid in ACN). The injection volume was 10 μL, and the column oven temperature was 40°C. The flow rate was 0.25 mL/min with a linear gradient elution over 15 min. The eluting gradient was as follows: 0.0–1.0 min (0% solvent B), 1.0–8.0 min (0–100% solvent B), 8.0–11.0 min (100% solvent B), 11.0–12.0 min (100–0% solvent B), and 12.0–15.0 min (0% solvent B). A blank sample was analyzed after each sample to avoid any carry-over effects. Additionally, a quality control sample was set up at five-sample intervals for peak area normalization and to monitor the stability of the system. The electrospray was operated in positive and negative ionization modes with a default data-dependent acquisition method. In addition, an MS full scan was performed in profile mode at 60,000 resolutions, followed by MS2 scans at 15,000 resolutions. The scan range was set from 70 to 1,000 *m*/*z*. Electrospray ionization source conditions were set as follows: spray voltage was 3.5 kV for positive mode and −3.5 kV for the negative; normalized collision energy was 25; the capillary temperature was 280°C; whereas sheath and aux gas were 30 and 5 arbitrary units, respectively. Raw data files were converted into mzML format using ProteoWizard and processed via R package XCMS (version 3.2). A data matrix composed of mass-to-charge ratio (*m*/*z*), retention time, and peak intensity values was generated after preprocessing. Each peak was annotated using an in-house MS2 database. The score cutoff for annotation was set at 0.5. The ion peak of missing values <5% of all samples in each metabolite was filled with half of the minimum intensity value. All identified metabolites were transformed to log_10_ area intensity for downstream analysis.

### 2.9 Correlation analysis

All correlation analyses were assessed using Spearman’s rank correlation analysis, and *p* < 0.05 was considered significant. To reflect the relationship between the original abundance information of bacterial taxa, rarefied log_10_-transformed ASV abundances of species-level bacterial taxa were estimated in the correlation analysis. ASV abundance information was rarefied to a sequence depth reaching stable observed features in each sample. Only the species-level bacterial taxa with a prevalence >25% in all participants were used in the correlation analysis. The correlation network was visualized through the R program (ggraph and ggcor packages) and Cytoscape (Version 3.10.0, http://www.cytoscape.org). The correlation heat map of bacterial species and serum metabolites was generated using GraphPad Prism v10.4.1 software (San Diego, CA, United States).

### 2.10 Statistical analyses

To evaluate longitudinal changes in kidney function markers—creatinine (CRE), blood urea nitrogen (BUN), symmetric dimethylarginine (SDMA), and gut-derived uremic toxins (GDUTs: TMAO, PCS, IS, and PS) across all 14 cats, linear mixed-effects models (LMM) were applied, with time as a fixed effect and cat ID as a random effect. Estimated marginal means with 95% confidence intervals (CIs) were reported, and statistical significance was set at *p* < 0.05. For short-chain fatty acids (SCFAs: acetate, propionate, and butyrate), comparisons between 0 and 8 weeks were performed using paired *t*-tests (for parametric data) or Wilcoxon signed-rank tests (for non-parametric data), following normality testing with the Shapiro–Wilk test. The same statistical approach was applied to timepoint comparisons within the high responder (HR) subgroup (*n* = 5), given the small sample size and paired structure of the data. For omics-based analyses, including microbial biomarkers, gut microbial functions, and serum metabolites, the Wilcoxon signed-rank test was used for paired comparisons, and the Wilcoxon rank-sum test for unpaired comparisons, based on relative abundance or log₁₀-transformed peak intensity data. All statistical tests were two-tailed and performed using SPSS (IBM SPSS Statistics v23) and GraphPad Prism (v10.4.1), with *p* < 0.05 considered statistically significant. *p*-values between 0.05 and 0.10 were interpreted as indicative of statistical trends.

Given the small sample size and exploratory nature of this pilot study, we acknowledge the potential for type II errors and that some effects of Lm intervention may be underestimated (43). Confidence intervals were used to assess the distribution and potential clinical relevance of observed changes (44, 45). Findings with *p*-values between 0.05 and 0.10 were interpreted as statistical trends, consistent with the criteria defined above, and were presented to highlight potential biological patterns of interest that may warrant further investigation in future studies. No corrections for multiple comparisons were applied; thus, results should be interpreted with appropriate caution.

## 3 Results

### 3.1 Study population

**Figure 1:**
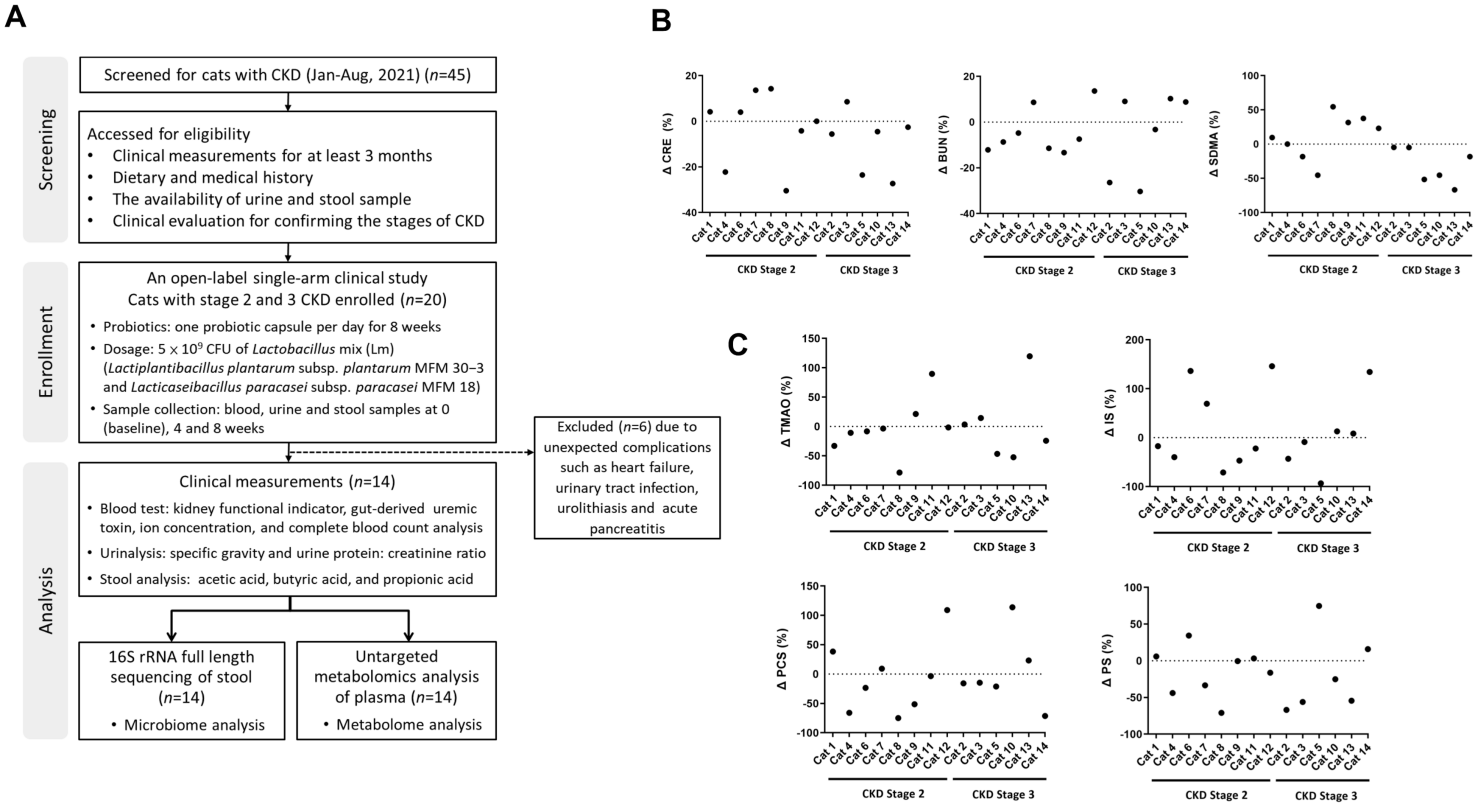
The study design showing kidney functional indicators and gut-derived uremic toxins (GDUTs) of cats with CKD. **(A)** Flow diagram of the study design and participating cats with CKD. The percentage of **(B)** serum kidney functional indicators and **(C)** GDUTs change before and after 8 weeks of Lm intervention. Δ (%) = 100 × (post-treatment value − baseline value)/baseline value.

The clinical application of Lm in CKD progression was evaluated in 20 cats. Here, 45 cats with CKD were initially underwent a comprehensive evaluation to obtain previous clinical measurements, dietary and medical histories, availability of clinical samples, and participation consent from their owners. The CKD stage and thyroxine levels of candidate cats were confirmed to ensure accurate assessment of kidney function before participation in the trial. Following a thorough assessment, only 20 cats with CKD met the criteria for enrollment, and 14 completed the study. Six were withdrawn from treatment due to unexpected complications, including heart failure, urinary tract infection, urolithiasis, and acute pancreatitis, which may influence the evaluation of kidney function. The study design and demographic characteristics of the 14 cats were shown in Figure 1A and Supplementary Table S1, respectively. The ratio of male to female cats was 4:3, with a median age of 12 years (range 4–17 years) and a median weight of 4.7 kg (range 3.1–8.3 kg). Among the 14 cats, eight had CKD stage 2 and six had stage 3.

### 3.2 The impact of Lm on the reduction of kidney functional indicator and GDUTs in serum

To evaluate changes in kidney function indicators and GDUTs over time, we performed linear mixed-effects model (LMM) analyses with time as a fixed effect and cat ID as a random effect. Estimated marginal means (EM Means) with their 95% confidence intervals (CIs) were presented in Table 1. No statistically significant differences were observed across the three time points for serum CRE, BUN, or SDMA (*p* > 0.05), nor for the GDUTs TMAO, IS, PCS, or PS (*p* > 0.05). Additionally, effect sizes (partial eta-squared, *η*^2^) for the time effect were small across all markers (range: 0.01–0.09), further supporting the lack of statistically significant change over the study period (data not shown). Although no significant time effects were detected, descriptive evaluation of the EM Means and CIs indicated a general tendency toward lower distributions across all markers after the 8-week intervention period. Raw descriptive statistics for serum kidney function indicators and GDUTs at each time point were presented in Supplementary Table S2. Additionally, descriptive review of individual trajectories indicated that approximately 57–64% of cats exhibited a tendency toward stabilization or reduction in these markers by week 8, although these changes were not statistically significant (Figures 1B,C).

**Table 1:** Estimated marginal means, confidence intervals, and overall time effects for serum kidney function indicators and gut-derived uremic toxins in cats with CKD before, during, and after Lm intervention.

Category	Indicators	Before Lm intervention	In Lm intervention	After Lm intervention	*p*-value
		0W	4W	8W	Overall
Kidney function indicator	CRE (mg/dL)	1.94 (−0.65 to 4.53)	1.82 (−0.52 to 4.15)	1.78 (0.21–3.35)	0.71
	BUN (mg/dL)	25.14 (−15.28 to 65.56)	23.93 (−16.26 to 64.12)	22.86 (−17.39 to 63.10)	0.87
	SDMA (μg/dL)	14.72 (8.81–20.64)	10.29 (4.32–16.27)	12.08 (6.39–17.76)	0.34
Gut-derived uremic toxin	TMAO (ppb)	198.7 (−1406.7 to 1804.0)	28.8 (−718.6 to 776.2)	69.6 (−1334.9 to 1474.0)	0.64
	PCS (ppb)	6775.5 (2565.3–10985.7)	7688.4 (3183.5–12193.3)	6076.6 (1896.4–10256.9)	0.58
	IS (ppb)	1188.9 (−935.9 to 3313.6)	1390.5 (−697.3 to 3478.4)	722.6 (−1349.7 to 2794.9)	0.80
	PS (ppb)	297.0 (−242.6 to 836.6)	682.2 (−672.2 to 2036.6)	95.9 (−320.7 to 512.5)	0.49

Data were presented as estimated marginal means with 95% confidence intervals. *p*-values for overall time effects were derived from linear mixed-effects model (LMM) analyses using type III test of fixed effects. Pairwise comparisons between time points were performed with Bonferroni correction; all *p*-values were >0.05 and were not shown here.

0W: baseline before Lm intervention; 4W: 4-week Lm intervention; 8W: 8-week Lm intervention.

CRE, creatinine; BUN, blood urea nitrogen; SDMA, symmetric dimethylarginine; TMAO, trimethylamine-N-oxide; IS, indoxyl sulfate; PCS, *p*-cresyl sulfate; PS, phenyl sulfate.

Additional linear regression analysis of the relationship between serum creatinine and TMAO over the intervention period revealed a progressive flattening of the correlation slope and a weakening of their correlation. At baseline (week 0), creatinine and TMAO were strongly correlated (*r* = 0.75, *p* = 0.002). This correlation remained moderate after 4 weeks of Lm intervention (*r* = 0.66, *p* = 0.011), but was noticeably weaker and no longer statistically significant after 8 weeks (*r* = 0.37, *p* = 0.196). These findings suggested a temporal dissociation between TMAO and creatinine during the course of Lm administration. While creatinine levels remained relatively stable, the weakening correlation may reflect a more pronounced reduction in TMAO, supporting the hypothesis that Lm intervention may modulate gut-derived uremic toxin levels independently of creatinine dynamics (Supplementary Figure S1). Serum and urine biochemical parameters were stabilized during Lm intervention, indicating no adverse effects of Lm in cats with CKD (Supplementary Table S3).

### 3.3 Lm intervention changed the population of specific bacterial species in cats with CKD

**Figure 2:**
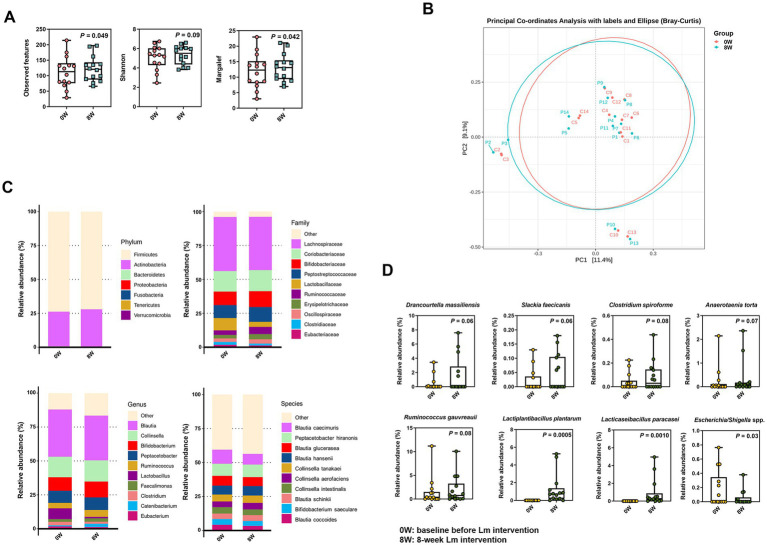
Microbial composition in cats with CKD before and after *Lactobacillus* mix (Lm) intervention. **(A)** Alpha-diversity indices. **(B)** PCoA plot reflecting beta diversity (Bray–Curtis) of individual cats with CKD. **(C)** The most abundant bacterial phyla, family, genera, and species. **(D)** Relative abundance of bacterial species enriched or diminished after Lm intervention. 0W: baseline before Lm intervention; 8W: 8-week Lm intervention. The matched-paired Wilcoxon signed-rank test was used.

We performed fecal full-length 16S rRNA gene sequencing analysis in all cats with CKD to explore the link between Lm intervention and the gut microbiota composition. As shown in Figure 2A, alpha diversity indices (observed features and Margalef’s richness) significantly increased after Lm intervention (*p* < 0.05), indicating enhanced microbial richness. Additionally, the Shannon index showed a trend toward increase (*p* = 0.09), suggesting a potential shift in both richness and evenness.

In contrast, beta diversity displayed in the PCoA plot revealed only a minor shift in overall gut microbiota configuration following Lm intervention, accounting for 11.4 and 9.1% of the variance along PC1 and PC2, respectively (Figure 2B). These results suggested that while Lm intervention increased microbial richness, it induced only modest compositional changes and did not significantly alter the overall structure of the core gut microbiota.

Our analysis of the gut microbial configuration of all tested cats with CKD demonstrated considerable differences before and after Lm intervention at varying levels (Figure 2C). *Firmicutes* and *Actinobacteria* were the most prevalent phyla in the gut microbiota of cats with CKD, accounting for >99.5% of the total population. Furthermore, an increase in the relative abundance of *Actinobacteria* was observed following Lm intervention. At the family level, the gut microbiota comprised 10 prominent families. *Lachnospiraceae* was the most dominant family (39.6%), followed by *Coriobacteriaceae* (15.3%), *Bifidobacteriaceae* (10.9%), *Peptostreptococcaceae* (10.2%), *Lactobacillaceae* (6.4%), *Ruminococcaceae* (4.3%), *Erysipelotrichaceae* (3.2%), *Oscillospiraceae* (2.9%), *Clostridiaceae* (1.8%), and *Eubacteriaceae* (1.5%) within the overall cat population before and after Lm intervention. *Blautia* was the most abundant at the genus level (33.9%), followed by *Collinsella* (15.3%), *Bifidobacterium* (10.9%), *Peptacetobacter* (9.0%), *Ruminococcus* (4.6%), *Lactobacillus* (4.5%), *Faecalimonas* (2.2%), *Clostridium* (2.0%), *Catenibacterium* (1.7%), and *Eubacterium* (1.5%).

A total of 196 bacterial species were identified in the gut microbiota of the cats. The top 10 bacterial species were *Blautia caecimuris* (9.1%), *Peptacetobacter hiranonis* (9.0%), *Blautia glucerasea* (6.9%), *Blautia hansenii* (6.8%), *Collinsella tanakaei* (5.2%), *Collinsella aerofaciens* (4.5%), *Collinsella intestinalis* (4.4%), *Blautia schinkii* (4.2%), *Bifidobacterium saeculare* (3.9%), and *Blautia coccoides* (3.8%). A trend toward an increase in the abundance of five identified species was observed, including *Drancourtella massiliensis* (*p* = 0.06), *Slackia faecicanis* (*p* = 0.06), *Clostridium spiroforme* (*p* = 0.08), *Anaerotaenia torta* (*p* = 0.07), and *Ruminococcus gauvreauii* (*p* = 0.06). In contrast, *Escherichia*/*Shigella* spp. showed a statistically significant decrease (*p* = 0.03) following Lm intervention (Figure 2D and Supplementary Table S4). Moreover, *L. plantarum* and *L. paracasei*, the two species comprising the Lm probiotics, were enriched in most cats with CKD after the Lm intervention but were not detected in the gut microbiota prior to intervention. The relative abundance of both strains significantly increased after Lm intervention (*p* < 0.01) (Figure 2D).

### 3.4 Lm intervention altered the gut microbial function

**Figure 3:**
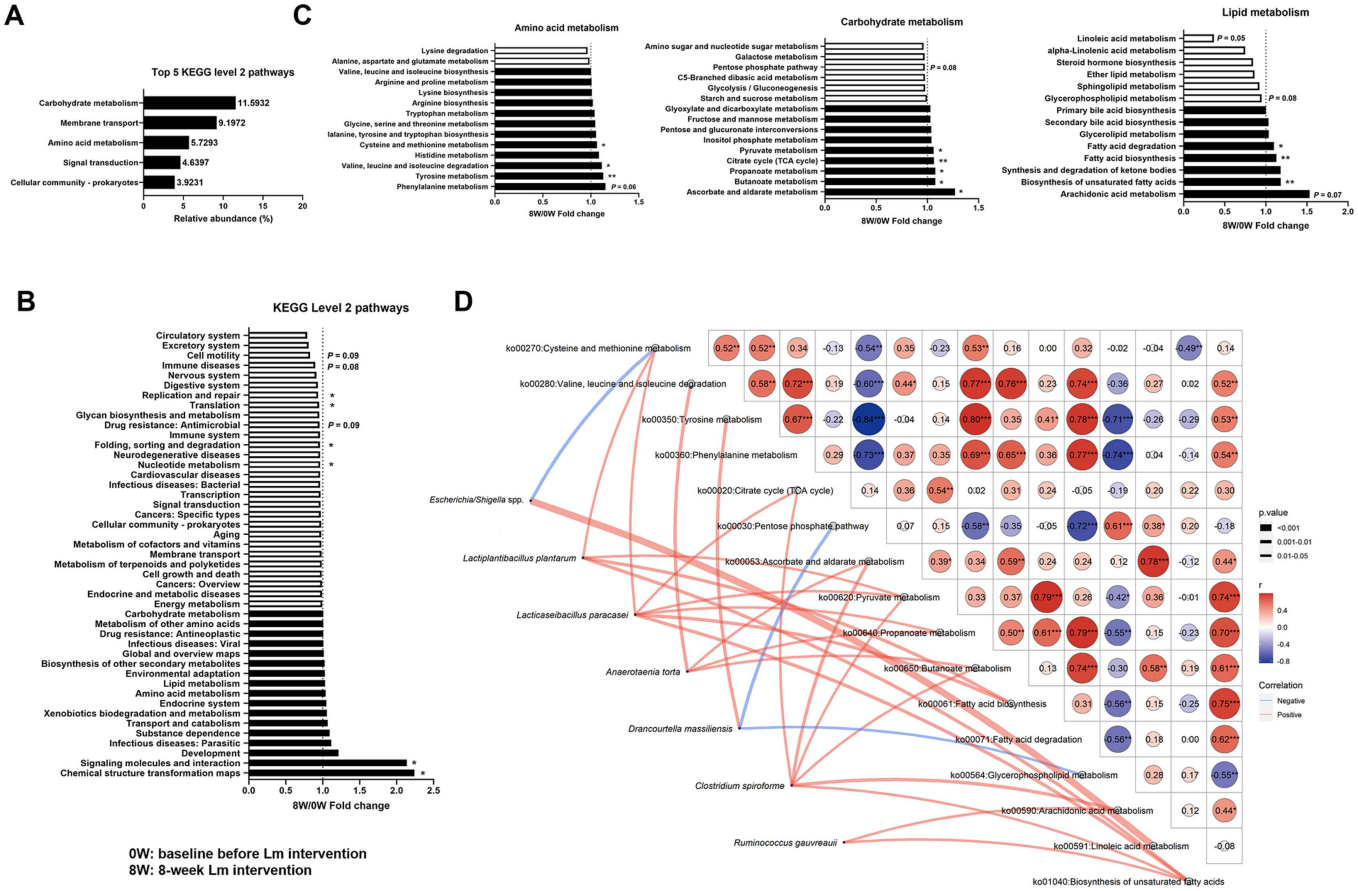
Gut microbial functions identified in cats with CKD. **(A)** The five most abundant KEGG level 2 pathways. **(B)** Fold change of KEGG level 2 pathways after *Lactobacillus* mix (Lm) intervention. **(C)** Fold change of KEGG level 3 pathways in amino acid, carbohydrate, and lipid metabolism after Lm intervention, as determined through the matched-paired Wilcoxon signed-rank test. Fold change = post-treatment (8W)/baseline (0W). 0W: baseline before Lm intervention; 8W: 8-week Lm intervention. **(D)** Spearman’s correlation analysis of bacterial species and KEGG level 3 pathways. ^*^p < 0.05 and ^**^p < 0.01.

Predicted KEGG profiles were used to determine gut microbial functions. Here, 309 third-level pathways belonging to six first-level categories (“Metabolism,” “Environmental Information Processing,” “Genetic Information Processing,” “Cellular Processes,” “Human Diseases,” and “Organismal Systems”) were recognized as involving feline gut microbial metabolic pathways (data not shown). Forty-five categories were observed at the second level, with “Carbohydrate metabolism,” “Membrane transport,” “Amino acid metabolism,” “Signal transduction” and “Cellular community—prokaryotes,” as the most abundant (Figure 3A). The identified KEGG pathways were compared after Lm intervention. Two second-level pathways (“Chemical structure transformation maps” and “Signaling molecules and interaction”) showed significant upregulation, and four pathways (“Nucleotide metabolism,” “Folding, sorting and degradation,” “Translation,” and “Replication and repair”) demonstrated significant downregulation in the gut microbiomes (*p* < 0.05; Figure 3B). Additionally, three second-level pathways (“Drug resistance: Antimicrobial,” “Immune diseases,” and “Cell motility”) showed a trend toward decreased abundance, with *p*-values ranging from 0.08 to 0.09. Eleven downstream functions involving amino acid, carbohydrate, and lipid metabolism (tyrosine metabolism, valine, leucine and isoleucine degradation, cysteine and methionine metabolism, ascorbate and aldarate metabolism, butanoate metabolism, propanoate metabolism, citrate cycle, pyruvate metabolism, biosynthesis of unsaturated fatty acids, fatty acid biosynthesis, and fatty acid degradation) were significantly enriched after Lm intervention (*p* < 0.05; Figure 3C). Additionally, two pathways (phenylalanine metabolism and arachidonic acid metabolism) showed a trend toward increased relative abundance, while three pathways (pentose phosphate pathway, glycerophospholipid metabolism, and linoleic acid metabolism) exhibited a trend toward decreased relative abundance (*p* = 0.05–0.08; Figure 3C).

A Spearman’s correlation network was constructed between eight bacterial species and 16 KEGG level 3 pathways that were significantly different before and after the Lm intervention. This network construction aimed to identify the effects of gut bacterial species involved in GDUTs and SCFAs production in the gut microbiota. Eight bacterial species exhibited 24 positive and 3 negative associations with KEGG level 3 pathways (*p* < 0.05, Figure 3D). Four species (*L. plantarum*, *L. paracasei*, *A. torta*, and *C. spiroforme*) were positively correlated with four SCFAs biosynthetic pathways, namely the citrate cycle, pyruvate metabolism, propanoate metabolism, and butanoate metabolism. Similarly, the two species comprising the Lm probiotics were strongly correlated with SCFAs biosynthetic pathways, whereas *D. massiliensis* exhibited a negative association with the pentose phosphate pathway. ‘Only *D. massiliensis* was positively correlated with tyrosine metabolism.

### 3.5 Lm intervention altered serum metabolomic profiles

**Figure 4:**
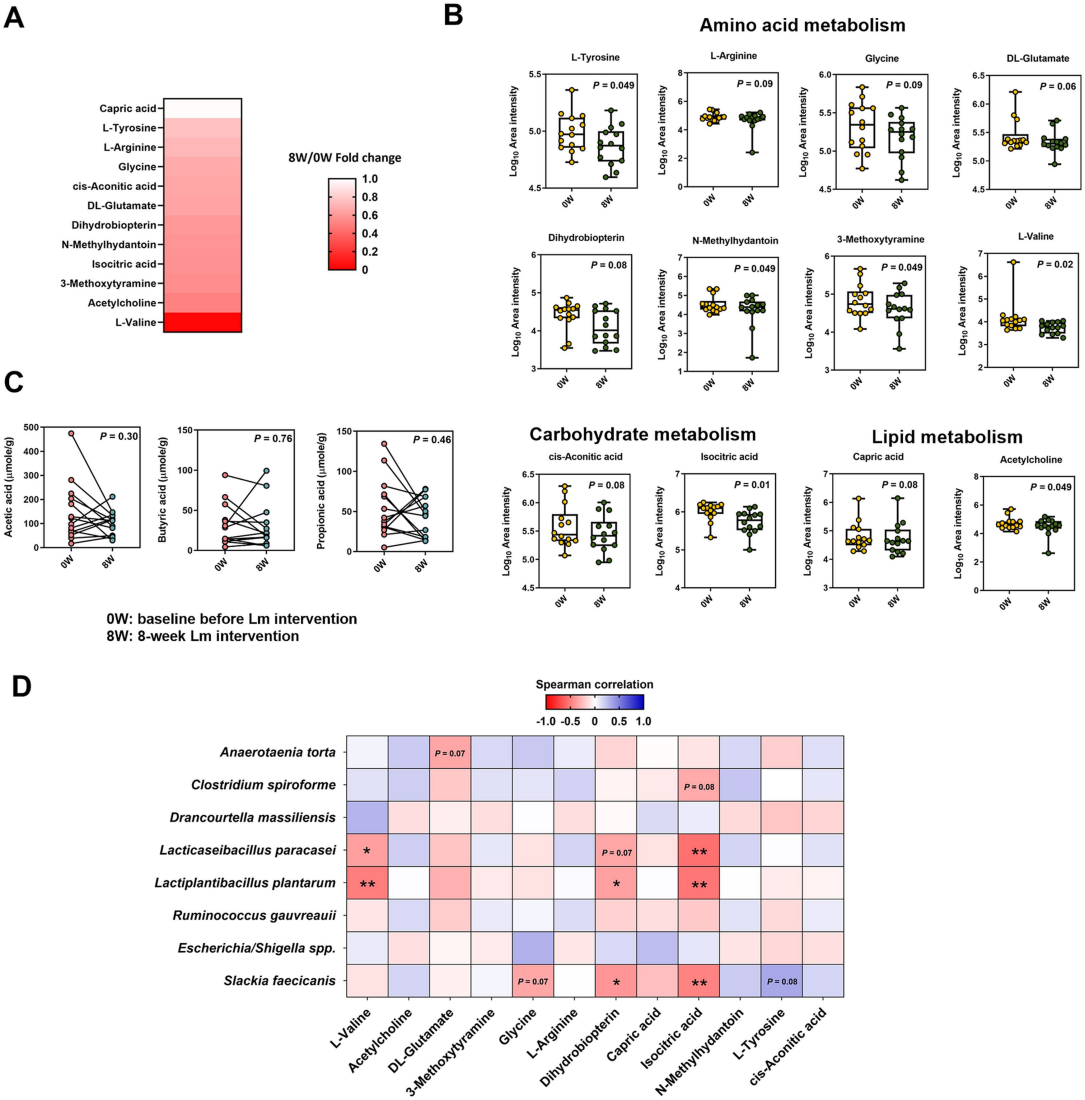
Metabolome composition in cats with CKD before and after *Lactobacillus* mix (Lm) intervention. **(A)** Fold change of serum metabolites showing significant change after Lm intervention (*p* < 0.1). Fold change = post-treatment (8W)/baseline (0W). **(B)** Log_10_ area intensity of serum metabolites diminished after Lm intervention. **(C)** Concentration of fecal short-chain fatty acids. 0W: baseline before Lm intervention; 8W: 8-week Lm intervention. The matched-paired Wilcoxon signed-rank test was used. **(D)** Spearman’s correlation analysis of bacterial species and serum metabolites. ^*^p < 0.05 and ^**^p < 0.01.

To clarify the relationship between the gut microbiome associated with Lm intervention and the serum metabolome, we profiled the serum metabolites using untargeted ultra-high-performance liquid chromatography coupled with a high-resolution Orbitrap Elite mass spectrometer (UHPLC-HRMS), detecting 6,372 and 4,454 variables under positive and negative ion modes, respectively. After annotation, 235 metabolites were identified in serum samples (data not shown). Twelve metabolites related to amino acid (L-valine, DL-glutamate, 3-methoxytyramine, glycine, L-arginine, N-methylhydantoin, L-tyrosine, and dihydrobiopterin), carbohydrate (isocitric acid and cis-aconitic acid), and lipid metabolism (capric acid and acetylcholine) showed either statistically significant decreases (*p* < 0.05) or trends toward reductions (*p* = 0.06–0.09) following Lm intervention (Figures 4A,B). In addition, the absolute levels of acetate, propionate, and butyrate in feline feces (Figure 4C) showed no significant differences before and after Lm intervention (*p* > 0.05).

Spearman’s rank correlation between the eight gut bacterial taxa and 12 serum metabolites revealed seven significant correlations (*ρ* < −0.37, *p* < 0.05) (Figure 4D). Negative correlations were primarily observed between two Lm species (*L. plantarum* and *L. paracasei*) and three metabolites (L-valine, dihydrobiopterin, and isocitric acid). *S. faecicanis*, a bacterial species, also showed a negative correlation with dihydrobiopterin, and isocitric acid.

### 3.6 The abundance of two probiotic species in the intestinal tract affects the efficacy of Lm intervention in managing CKD

**Figure 5:**
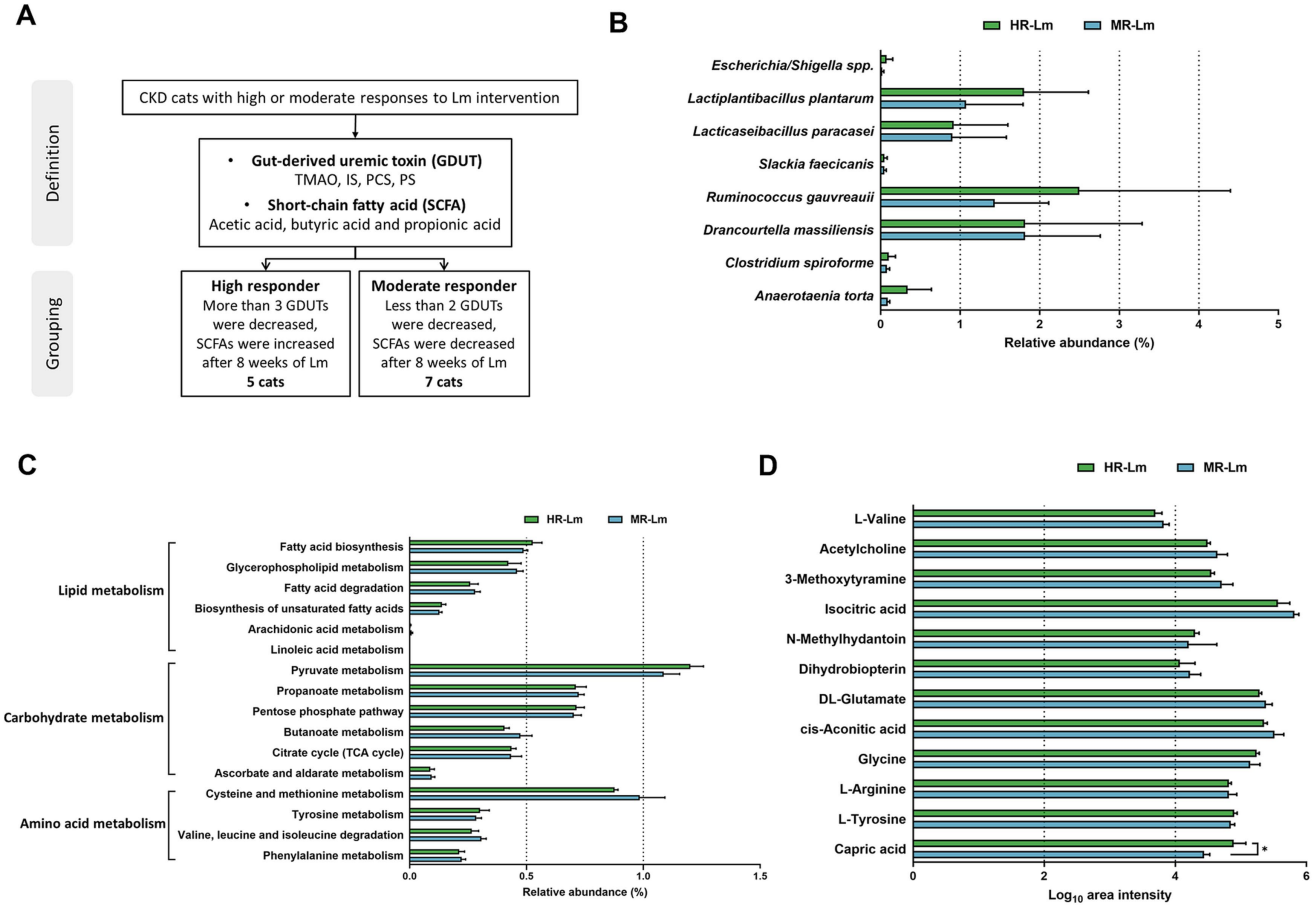
The abundance of differential microbial biomarkers, gut microbial functions, and serum metabolites in high responder (HR) and moderate responder (MR) after *Lactobacillus* mix (Lm) intervention. **(A)** The grouping criteria for HR and MR cats with CKD. **(B)** Bacterial species, **(C)** Gut microbial functions, and **(D)** Serum metabolites after 8 weeks of Lm intervention, as determined through the Wilcoxon rank-sum test. HR-Lm or MR-Lm: results after 8 weeks of Lm intervention in HR or MR cats with CKD, respectively. ^*^p < 0.05.

To further investigate how changes in microbial derived metabolites were associated with gut microbial features, we conducted an exploratory subgroup analysis to evaluate individual variability in response to Lm intervention among cats with CKD. Cats were classified as high responders (HR) if they exhibited reductions in at least three GDUTs and increases in all measured SCFAs following the intervention. Cats showing reductions in fewer than two GDUTs and decreases in all SCFAs were categorized as moderate responders (MR). Two cats who did not clearly meet the criteria for either group were excluded from the subgroup comparison. This classification was performed *post hoc* and was intended to generate hypotheses regarding microbial biomarkers, microbial functions, and metabolite signatures associated with differential biological responses to the intervention. Based on these criteria, five cats were categorized as HR and seven as MR (Figure 5A and Table 2).

**Table 2:** Serum kidney functional indicators, gut-derived uremic toxins, and short-chain fatty acid levels in high responder (HR) before and after *Lactobacillus* mix (Lm) intervention.

Category	Indicators	Before Lm intervention	After Lm intervention	*p*-value
		0W	8W	0W vs. 8W
Kidney functional indicator	CRE (mg/dL)	3.02 (2.09–3.95)	2.88 (1.72–4.04)	0.43
	BUN (mg/dL)	35.40 (21.87–48.93)	30.00 (20.22–39.78)	0.12
	SDMA (μg/dL)	18.80 (6.94–30.66)	16.20 (10.83–21.57)	0.70
Gut-derived uremic toxin	TMAO (ppb)	1165.0 (109.8–2220.0)	762.5 (156.2–1369.0)	0.24
	PCS (ppb)	5843.0 (687.1–10999.0)	2723.0 (1455.0–3991.0)	0.08
	IS (ppb)	3590.0 (93.3–7087.0)	1173.0 (387.6–1958.0)	0.09
	PS (ppb)	1216.0 (−179.3 to 2610.0)	701.7 (−109.1 to 1512.0)	0.12
Short-chain fatty acid	AA (μmole/g)	50.85 (20.24–81.45)	92.64 (40.11–145.20)	0.008
	PA (μmole/g)	25.79 (8.47–43.12)	50.66 (14.32–86.99)	0.02
	BA (μmole/g)	15.09 (3.07–27.10)	32.66 (−14.05 to 79.36)	0.06

Data were presented as mean (95% confidence intervals). Variables were tested by the paired *t*-tests or the matched-paired Wilcoxon signed-rank test.

0W: baseline before Lm intervention; 8W: 8-week Lm intervention.

CRE, creatinine; BUN, blood urea nitrogen; SDMA, symmetric dimethylarginine; TMAO, trimethylamine-N-oxide; IS, indoxyl sulfate; PCS, *p*-cresyl sulfate; PS, phenyl sulfate; AA, acetic acid; BA, butyric acid; PA, propionic acid.

We first examined the differences between the HR and MR groups in previously identified microbial biomarkers, microbial functions, and serum metabolites. Analyses considered both statistically significant differences (*p* < 0.05) and biologically relevant trends (0.05 < *p* < 0.1) before and after Lm intervention (Supplementary Table S5). Although no microbial species or KEGG pathways showed statistically significant differences between groups, the HR group was relatively enriched in eight bacterial species (Figure 5B). These cats also showed elevated microbial functions related to tyrosine metabolism, the citrate cycle, the pentose phosphate pathway, pyruvate metabolism, linoleic acid metabolism, biosynthesis of unsaturated fatty acids, and fatty acid biosynthesis (Figure 5C). In terms of metabolites, capric acid levels were significantly higher in HR cats than in MR cats, while eight additional metabolites, excluding L-tyrosine, glycine, and N-methylhydantoin, were lower in the HR group (Figure 5D).

**Figure 6:**
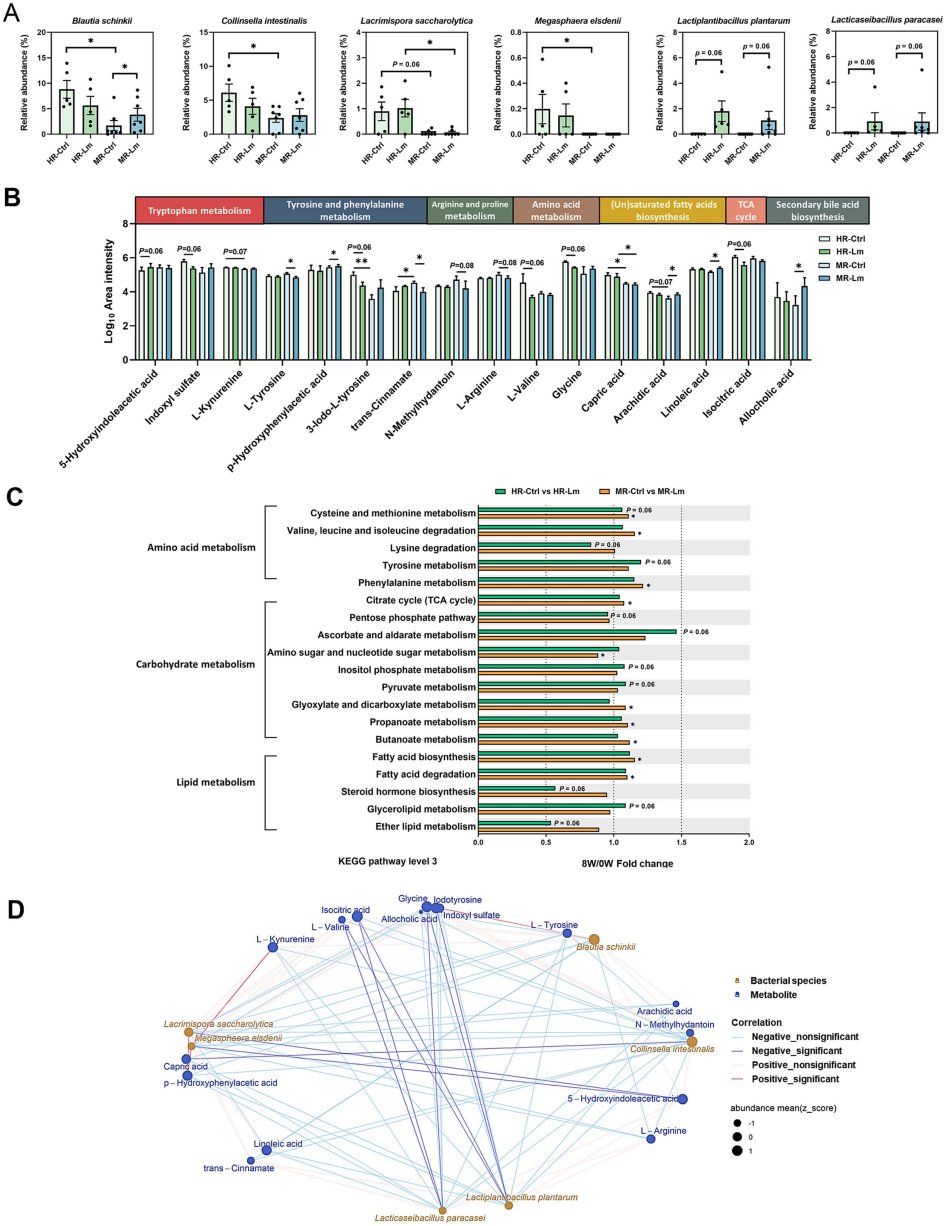
Differential microbial biomarkers, gut microbial functions, and serum metabolites in high responder (HR) and moderate responder (MR) before and after *Lactobacillus* mix (Lm) intervention. **(A)** Relative abundance of differential microbial biomarkers. **(B)** Log_10_ area intensity of differential serum metabolites. **(C)** Fold change of differential KEGG level 3 pathways involved in amino acid, carbohydrate, and lipid metabolism. The Wilcoxon rank-sum and signed-rank tests were utilized to compare unpaired and paired cats with CKD, respectively. HR-Ctrl or MR-Ctrl: the baseline in HR or MR cats before Lm intervention, respectively. HR-Lm or MR-Lm: results after 8 weeks of Lm intervention in HR or MR cats, respectively. ^*^p < 0.05 and ^**^p < 0.01. **(D)** Spearman’s correlation analysis of bacterial species and serum metabolites in HR cats.

Further pairwise comparisons among subgroups (HR-Control vs. MR-Control, HR-Control vs. HR-Lm, and MR-Control vs. MR-Lm), with *p* < 0.1 as selection criteria, identified four additional microbial biomarkers (six in total) (Figure 6A) and nine serum metabolites (16 in total) (Figure 6B) that differed between groups. Three bacterial taxa, *Lacrimispora saccharolytica* and two Lm strains (*L. plantarum* and *L. paracasei*), were increased in both the HR and MR groups, with higher relative abundance in the HR group after Lm intervention. In contrast, *B. schinkii* and *C. intestinalis* decreased in the HR group and increased in the MR group (Figure 6A). Differences were also noted across 19 KEGG level 3 pathways, involving amino acid, carbohydrate, and lipid metabolism (Figure 6C).

Of the 16 serum metabolites, 10 showed opposite tendencies following Lm intervention. Seven metabolites, including IS, L-kynurenine, 3-iodo-L-tyrosine, *p*-hydroxyphenylacetic acid, glycine, arachidic acid, and allocholic acid, showed a tendency to decrease in the HR group but increase in the MR group. Conversely, 5-hydroxyindoleacetic acid, *trans*-cinnamate, and L-arginine tended to increase in the HR group and decrease in the MR group. The remaining six metabolites exhibited similar tendencies in both groups. For instance, linoleic acid showed a tendency to increase across both groups, whereas L-tyrosine, N-methylhydantoin, L-valine, capric acid, and isocitric acid tended to decrease (Figure 6B).

To further examine potential host-microbe interactions, we analyzed Spearman’s rank correlations between the six microbial biomarkers and 16 serum metabolites in the HR group (Figure 6D). Fourteen significant correlations (|*ρ*| > 0.66, *p* < 0.05) were identified. Notably, *L. plantarum* and *L. paracasei* were negatively correlated with several amino acids and uremic toxins, while *Megasphaera elsdenii* showed negative correlation with 5-hydroxyindoleacetic acid and positive correlations with L-kynurenine and *p*-hydroxyphenylacetic acid.

**Figure 7:**
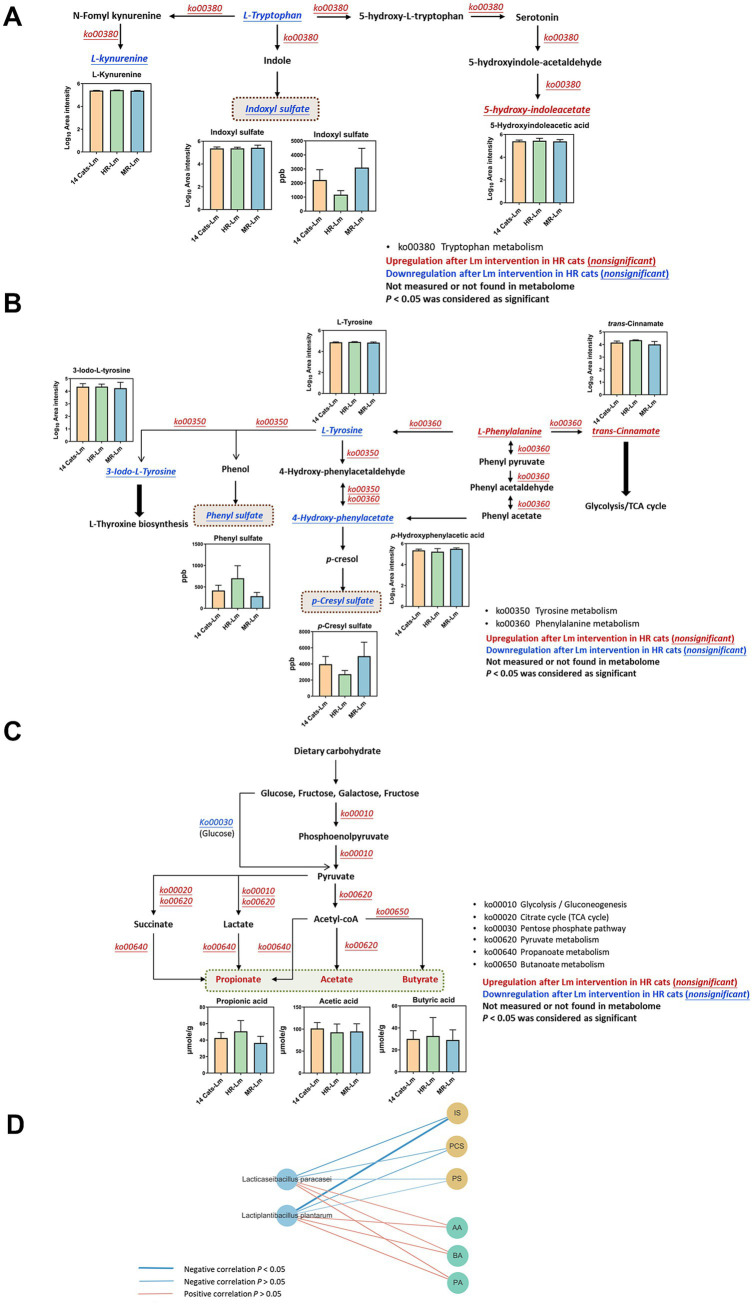
Regulation of metabolites and microbial functions involved in gut-derived uremic toxin (GDUTs) and short-chain fatty acid (SCFA) biosynthesis in HR cats after *Lactobacillus* mix (Lm) intervention. Metabolic pathways involved in **(A)** indoxyl sulfate, **(B)** *p*-cresyl sulfate and phenyl sulfate, and **(C)** SCFA biosynthesis. Fourteen cats-Lm, HR-Lm, or MR-Lm: results after 8 weeks of Lm intervention in 14 cats, HR cats, or MR cats, respectively. **(D)** Spearman’s correlation analysis of Lm strains and quantitated GDUTs/SCFAs concentration in HR cats.

Microbial functional pathways associated with IS, PS, and PCS biosynthesis pathways were evaluated. In HR cats, functional predictions suggested higher relative abundance of tryptophan metabolism (ko00380) and phenylalanine metabolism (ko00360), although these changes were not statistically significant (*p* > 0.05), while tyrosine metabolism (ko00350) showed a trend toward increased abundance (*p* = 0.06) following Lm intervention. These functional shifts coincided with reductions in L-tryptophan and L-tyrosine levels, along with decreased concentrations of circulating IS, PCS, and PS (Figures 7A,B and Table 2). Regarding SCFA biosynthesis, six KEGG level 3 pathways within carbohydrate metabolism were implicated. The pentose phosphate pathway showed a trend toward decreased relative abundance (*p* = 0.06), while four other pathways, including glycolysis, citrate cycle, propanoate metabolism, and butanoate metabolism, exhibited higher relative abundance following Lm intervention, although these differences were not statistically significant (*p* > 0.05). Pyruvate metabolism also showed a trend toward increase (*p* = 0.06), corresponding with elevated SCFA concentrations (*p* = 0.008–0.06) observed in HR cats after Lm intervention (Figure 7C and Table 2).

A comparison of serum GDUTs (IS and PCS) and fecal SCFAs (propionate and butyrate) among HR, MR, and the full CKD cohort indicated that HR cats had lower levels of GDUTs and higher SCFAs after Lm intervention. Correlation analysis further showed that *L. plantarum* and *L. paracasei* were negatively correlated with GDUTs (IS, PCS, and PS) (−0.70 < *ρ* < −0.31, 0.02 < *p* < 0.38) and positively correlated with SCFAs (acetate, propionate, butyrate) (0.42 < *ρ* < 0.52, 0.13 < *p* < 0.23) in HR cats (Figure 7D). While these findings suggest a relationship between probiotic colonization and modulation of microbial-derived metabolites, they should be interpreted cautiously and viewed as hypothesis-generating observations that warrant further validation in larger, controlled studies.

## 4 Discussion

This study evaluated the potential effect of Lm (*L. plantarum* subsp. *plantarum* MFM 30-3 and *L. paracasei* subsp. *paracasei* MFM 18) in an open-label, single-arm pilot study involving cats with stage 2–3 CKD. Through integration of the gut microbiome and serum metabolome analyses, we explored host-microbe interactions and biological shifts potentially associated with Lm intervention.

Consistent with our previous findings in an adenine-induced CKD mouse model (22), cats receiving Lm interventions showed a general tendency toward reduced GDUTs and serum markers of kidney function, such as CRE and BUN. These observations are broadly aligned with prior human clinical studies where probiotic supplementation reduced levels of GDUTs (e.g., IS, PCS, TMAO, and indoxyl glucuronide) and precursors of uremic toxins (indole, *p*-cresol, and phenol) (46–50). The most common species used for CKD clinical trials are *Lactobacillus acidophilus* (47, 48, 51), *Lactobacillus* (*Lacticaseibacillus*) *casei* (24, 46, 48, 51, 52), *Bifidobacterium longum* (47, 51, 53, 54), and *Bifidobacterium bifidum* (53–55), with numerous strains that differ from Lm species.

The concept of gut dysbiosis in CKD is well-established in human and rodent studies (13–15, 17). Gut dysbiosis in patients with CKD expands the imbalance of intestinal flora, increases the population of proteolytic bacteria, and decreases the number of saccharolytic bacteria, resulting in a higher generation of GDUTs (13, 15–17). A high abundance of bacterial families with urease, uricase, and indole, as well as *p*-cresol-forming enzymes, can further accelerate CKD progression by affecting the biosynthesis of uremic toxin molecules. Moreover, the strong link between CKD and gut microbial dysbiosis suggests that modification of the gut microbiota could diminish uremic toxin levels (56). Despite limited research on CKD-related gut dysbiosis in cats, a study found that lower alpha-diversity indices (Chao1, Shannon, and Observed OTUs) in cats with CKD compared to healthy cats (7), which showed similar findings to those of human. In addition, another study observed significant changes associated with varying dysbiosis severity in the feline intestinal microbiota. The ratio of Gram-negative to Gram-positive microflora increased, especially in genera belonging to the family *Enterobacteriaceae*, such as *Escherichia* spp., *Enterobacter* spp., *Citrobacter* spp., *Klebsiella* spp., and *Proteus* spp. Conversely, the abundance of the genera *Lactobacillus* and *Bifidobacterium* decreased depending on the severity of dysbiosis, suggesting more proteolytic bacteria and less saccharolytic in cats with intestinal dysbiosis (57). In our study, we demonstrated that Lm intervention appeared to support a shift toward a more favorable microbial composition, including increased saccharolytic families (e.g., *Bifidobacteriaceae* and *Ruminococcaceae*) and reduced abundance of proteolytic families (e.g., *Clostridiaceae* and *Enterobacteriaceae*). Among three downregulated families (*Clostridiaceae*, *Enterobacteriaceae*, and *Peptococcaceae*) after Lm intervention (Supplementary Table S6), *Clostridiaceae* and *Enterobacteriaceae*, which contain GDUTs precursor-producing enzymes, were more abundant in patients with end-stage renal disease than in healthy participants (15). *Peptococcaceae* correlate positively with serum hippuric acid concentrations (58).

We further analyzed bacteria in species level, eight bacterial species significantly differed before and after Lm intervention. *Escherichia/Shigella* spp. belonging to the family *Enterobacteriaceae* are associated with deteriorating CKD progression. These bacteria possess proteolytic capacity and indole production (15, 59, 60). *Ruminococcus* and *Clostridium* species utilize various carbon sources to produce SCFAs, mainly butyrate, which is important for intestinal homeostasis (61–63). Five additional microbial species were enriched after Lm intervention, except *L. plantarum* and *L. paracasei*. *D. massiliensis*, *S. faecicanis*, and *A. torta* are commensal bacteria found in humans, dogs, and rabbits (64–66).

There are ongoing investigations on feline intestinal microbiota and its association with diseases. To our knowledge, our study is the first to address how Lm intervention facilitates the transition from dysbiosis to a healthy gut environment, resulting in improved diversity, decreased proteolytic bacteria (*Escherichia*/*Shigella* spp.), and restoration of bacteria whose abundance has decreased in cats with CKD (*Slackia*).

Alterations in gut microbial composition were accompanied by significant changes in KEGG microbial functions, with phenylalanine, tyrosine, propanoate, and butanoate metabolism being the four pathways most associated with the effect of Lm in managing CKD. Numerous studies have reported a relationship between the gut microbiome and phenylalanine/tyrosine/butanoate metabolism in patients with CKD and a CKD-low-protein diet (67–69). Abnormal phenylalanine metabolism has also been observed in patients with diabetic kidney disease (58). However, few studies have revealed how probiotics affect downstream microbial functions in humans and cats with CKD. Lm intervention modulated the gut microbiome, leading to changes in functional modules related to phenylalanine, tyrosine, and butanoate metabolism, which were associated with corresponding shifts in serum levels of GDUTs (IS, PCS, and PS) and stool levels of SCFAs (acetate, propionate, and butyrate).

Alteration of gut microbial function in amino acid and carbohydrate metabolism after Lm intervention significantly reduced downstream amino acids (L-valine, glycine, DL-glutamate, L-arginine, and L-tyrosine), as well as uremic toxin- (N-methylhydantoin), dopamine- (3-methoxytyramine), neurotransmitter- (dihydrobiopterin), and citrate cycle-related metabolites (isocitric acid and cis-aconitic acid). Elevated N-methylhydantoin (70), 3-methoxytyramine (71), dihydrobiopterin (72), isocitric acid, and cis-aconitic acid levels (73) have been observed in patients with CKD and animal models. N-methylhydantoin is also a product of creatinine degradation by gut microbes (74). Additionally, reduced serum amino acid levels were strongly associated with the efficiency of dietary amino acid utilization by gut microbes in the intestine (75, 76), owing to Lm-improved gut homeostasis. The serum metabolomic profiles observed in this study were directionally consistent with the gut microbiome findings, showing a tendency toward improved markers associated with kidney clearance capacity.

There was considerable heterogeneity in the responses to probiotic treatment in cats with CKD. Serum GDUTs reduction and fecal SCFAs elevation were observed in some cats after Lm intervention, and better kidney function was maintained. In probiotic clinical studies, individual factors such as diet, age, physiological condition, immune response, and indigenous gut microbiota often considerably influence the efficacy of probiotic supplementation and lead to divergent outcomes (77). To explore individual variation in response to Lm, we performed a *post hoc* exploratory subgroup analysis based on changes in GDUTs and SCFAs. This stratified approach identified microbial and metabolic differences between high responders (HR) and moderate responders (MR). The specific microbial configurations and metabolite profiles of these cats could provide more precise insights into the mechanisms underlying Lm function. Four bacterial species showed distinct populations in the HR and MR groups before Lm intervention. *M. elsdenii*, associated more with diabetic kidney disease than type 2 diabetes (78), only existed in the HR group and was decreased by Lm. Downregulation of *L. saccharolytica* is involved in autism spectrum disorders via *p*-cresol-induced autistic-like behaviors in mice (79). An increase in *L. saccharolytica* was observed in both the HR and MR groups after Lm intervention, suggesting a potential beneficial effect in cats with CKD, though this change was not statistically significant. *B. schinkii* (80) and *C. intestinalis* (81) are commensal microbes found in humans, dogs, and lambs. However, there are no reports related to their pathophysiological relevance in CKD. The differences in microbial features between the HR and MR groups after Lm intervention indicated the complex nature of host-microbiota interactions, leading to diverse effects on bacteria or metabolites after the consumption of probiotics (82).

**Figure 8:**
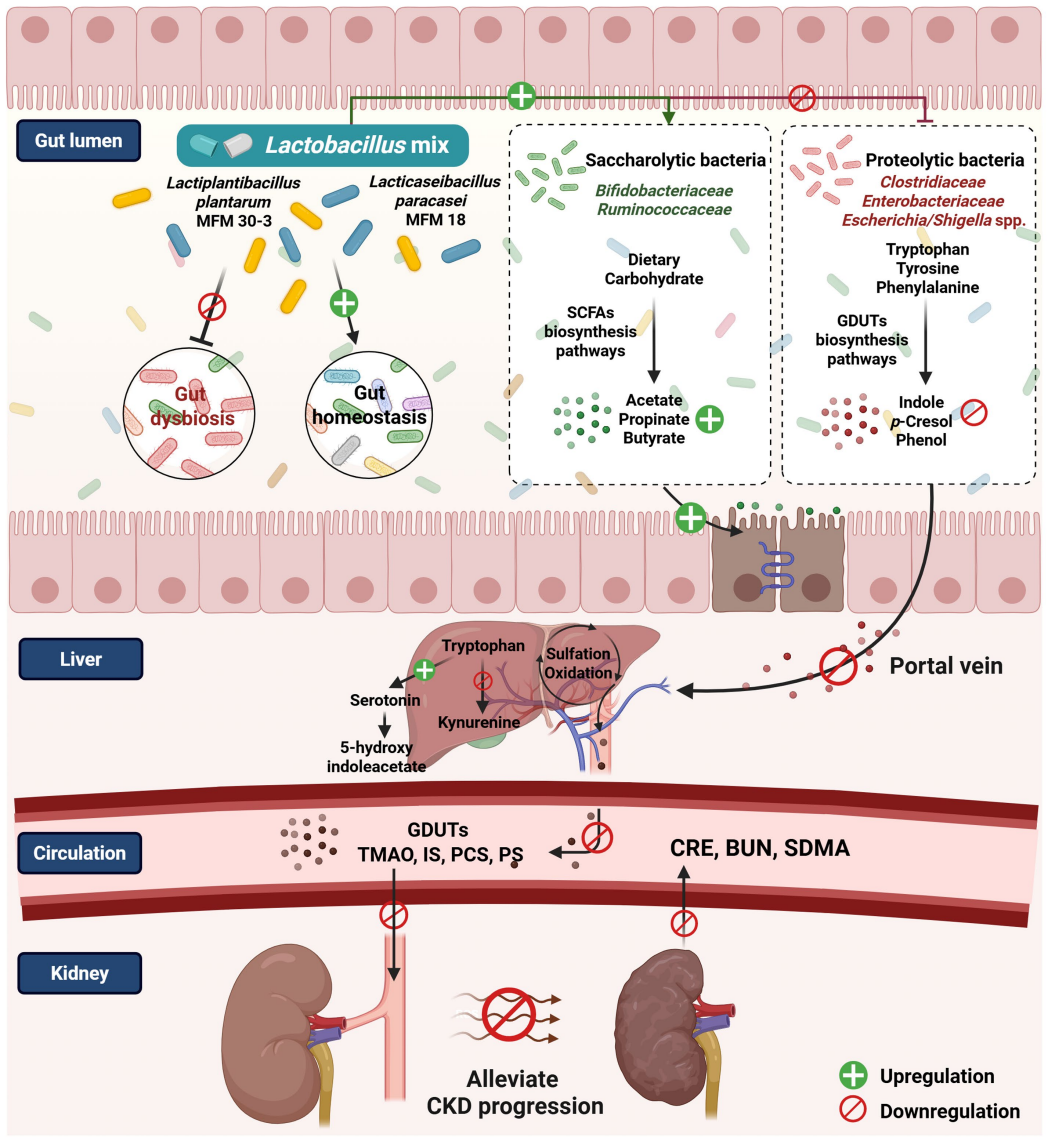
The proposed mechanism postulates that the *Lactobacillus* mix is associated with the gut microbiome and host metabolome in managing feline CKD (created with BioRender.com/fn0syc5).

Notably, *L. plantarum* and *L. paracasei* were detectable only after Lm intervention. Furthermore, their relative abundance was more abundant in the HR groups than in the MR cats. These personalized colonization patterns, alongside the presence of indigenous gut microbiota, suggest the possibility of host-specific colonization dynamics, possibly influenced by host factors such as variability in mucosal immune-related gene expression within the gastrointestinal tract (83). In addition, microbial competition, the timing of strain introduction (priority effects), and the absence or presence of related native species may further shape colonization outcomes (84, 85). Our combined gut microbial functions and serum metabolites revealed that levels of several key metabolites, associated with tryptophan metabolism (e.g., IS), tyrosine and phenylalanine metabolism (e.g., PCS and PS), and SCFAs biosynthesis (e.g., acetate, propionate, and butyrate), were linked to differences in Lm colonization and response profiles. These findings support the hypothesis that colonization efficiency may influence the modulation of microbial-derived metabolites and help explain differential biological responses to probiotic intervention. While preliminary, these findings suggest that the abundance of Lm strains may influence the modulation of gut-derived metabolites, underscoring the importance of future studies to clarify how host and microbial factors shape probiotic colonization dynamics and functional outcomes in the context of CKD (Figure 8).

Tryptophan metabolism can shift from serotonin synthesis toward kynurenine production in response to infection, stress, or alterations in the gut microbiota (86, 87). In the HR group, Lm intervention was associated with a trend toward increased levels of 5-hydroxyindoleacetic acid (*p* = 0.06) and decreased levels of IS (*p* = 0.06 based on metabolomics; *p* = 0.09 based on quantitative measurement). L-kynurenine levels also decreased, although not significantly (*p* = 0.19). Together, these findings suggested potential modulation of the tryptophan metabolism pathway. Lm intervention was associated with a decrease in the intermediate 4-hydroxy-phenylacetate, a precursor in the biosynthetic pathway of PCS (88), although this change was not statistically significant. In contrast, PCS levels in serum showed a trend toward reduction (*p* = 0.08). This result indicated that Lm shifted the metabolic pathway to prevent the production of toxic compounds. SCFA-related pathways (e.g., glycolysis, citrate cycle, propanoate, and butanoate metabolism) also showed increased activity in HR cats, aligning with elevated fecal SCFA levels. SCFAs have anti-inflammatory, anti-tumorigenic, and antimicrobial effects and maintain gut integrity. These properties indicate their importance in maintaining gut and immune homeostasis (89). While these patterns are consistent with proposed mechanisms of probiotic benefit, they should be interpreted within the exploratory scope of this pilot study.

This study has several limitations. First, it was conducted as an open-label, single-arm pilot trial without a control group, which limits the ability to differentiate true treatment effects from natural disease progression or time-related changes. Single-arm designs are commonly employed in early-stage exploratory research when minimal placebo effects are anticipated and when practical or ethical barriers make randomized controls difficult (90–93). The findings should be considered exploratory and intended to inform future hypothesis-driven studies. Second, our subgroup analysis, which categorized cats as high or moderate responders based on changes in gut-derived uremic toxins (GDUTs) and short-chain fatty acids (SCFAs), was performed *post hoc*. This classification was based on biologically relevant metabolite shifts and aimed to explore whether differences in microbial-derived metabolites were associated with distinct microbiome profiles and functional responses. While this approach provides valuable preliminary insights into gut-kidney interactions, it is exploratory and does not imply therapeutic efficacy. Third, the statistical methods for microbiome analysis were based on non-parametric Wilcoxon tests due to the pilot nature and sample size of this study. We acknowledge that compositional-aware methods (e.g., ANCOM-BC2, MaAsLin3) are more appropriate for microbiome data and will be implemented in future studies with larger cohorts to improve analytic rigor and control for confounders. Other limitations include the relatively small sample size, due to strict inclusion criteria and owner participation constraints, and the absence of a standardized diet, which may have contributed to variability in gut microbiota and metabolome profiles. Additionally, 16S rRNA sequencing was used, which limits the resolution of microbial functional characterization. Future work utilizing shotgun metagenomics, standardized dietary protocols, and randomized controlled designs will be critical to provide more comprehensive insights and to refine strategies for precision probiotic interventions in feline CKD.

## 5 Conclusion

This study presents the first integrated multi-omics analysis of probiotic intervention in feline CKD, demonstrating that Lm intervention improved gut dysbiosis and modulated gut microbial composition and functions related to the production of gut-derived uremic toxins and short-chain fatty acids. Subgroup analysis indicated that these functional changes were associated with favorable shifts in systemic metabolite profiles, including reduced levels of IS, PCS, and PS, and increased SCFAs. Additionally, individual variability in microbial metabolite responses may be linked to differences in Lm strain colonization. Collectively, these findings offer novel insights into the gut-kidney axis in cats and support the potential of probiotics as microbiota-targeted strategies for adjunctive CKD management.

## Data Availability

The data that support the findings of this study are available in NCBI Sequence Read Archive (SRA) at https://www.ncbi.nlm.nih.gov/bioproject/PRJNA987532, reference number PRJNA987532, and within the article and its Supplementary material.

## Glossary

ACN: Acetonitrile
ASVs: Amplicon sequence variants
BUN: Blood urea nitrogen
CCS: Circular consensus sequence
CKD: Chronic kidney disease
CRE: Creatinine
GDUTs: Gut-derived uremic toxins
HR: High responder
HRMS: High-resolution Orbitrap Elite mass spectrometry
IS: Indoxyl sulfate
KEGG: Kyoto Encyclopedia of Genes and Genomes
Lm: *Lactobacillus* mix
MR: Moderate responder
PCoA: Principal coordinates analysis
PCS: *p*-cresyl sulfate
PS: Phenyl sulfate
SCFAs: Short-chain fatty acids
SDMA: Symmetric dimethylarginine
TMAO: Trimethylamine-N-oxide
UHPLC: Ultra-high-performance liquid chromatography
UPC: Urine protein-to-creatinine ratio
