# Supplementary material for: Multi-omics analysis reveals the efficacy of two probiotic strains in managing feline chronic kidney disease through gut microbiome and host metabolome
Source: Front Vet Sci. 2025 Jun 18;12:1590388. doi: 10.3389/fvets.2025.1590388 (PMC12213445; doi:10.3389/fvets.2025.1590388)
Supplement: Supplementary file 1 [file Image_1.pdf]

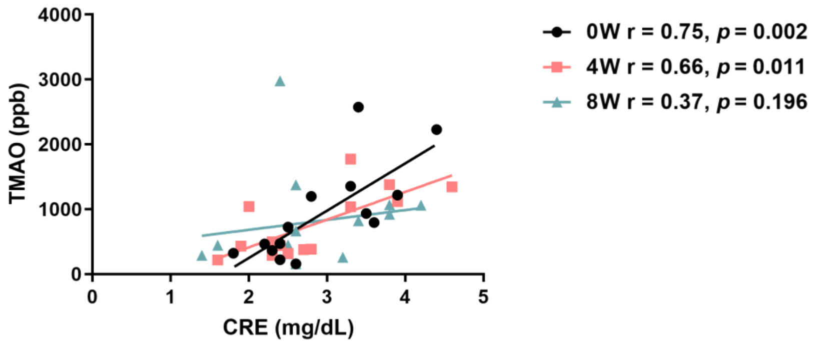


**Supplementary Figure S1.** Spearman's correlation of CRE and TMAO before, between, and after *Lactobacillus* mix (Lm) intervention. r represented Spearman's correlation coefficient. 0W: baseline before Lm intervention; 4W: 4-week Lm intervention; 8W: 8-week Lm intervention. CRE, creatinine; TMAO, trimethylamine-N-oxide.
