# Supplementary material for: Multi-omics analysis reveals the efficacy of two probiotic strains in managing feline chronic kidney disease through gut microbiome and host metabolome
Source: Front Vet Sci. 2025 Jun 18;12:1590388. doi: 10.3389/fvets.2025.1590388 (PMC12213445; doi:10.3389/fvets.2025.1590388)
Supplement: Supplementary file 2 [file Supplementary_file_1.docx]

**Supplementary Information**

**Additional file 1: Supplementary Table S1.** Demographic characteristics of study group. **Supplementary Table S2.** Serum kidney function indicators and gut-derived uremic toxins in cats with CKD before, during, and after Lm intervention. **Supplementary Table S3.** Serum and urine biochemical parameters of cats with CKD before, between, and after *Lactobacillus* mix (Lm) intervention. **Supplementary Table S4.** Bacterial species identified in cats with CKD before and after *Lactobacillus* mix (Lm) intervention. **Supplementary Table S5.** Selected microbial biomarkers, gut microbial functions, and serum metabolites in high responder (HR) and moderate responder (MR) after *Lactobacillus* mix (Lm) intervention. **Supplementary Table S6.** Bacterial families identified in cats with CKD before and after *Lactobacillus* mix (Lm) intervention.

**Additional file 2: Supplementary Figure S1.** Spearman’s correlation of CRE and TMAO before, between, and after *Lactobacillus* mix (Lm) intervention. r represented Spearman correlation coefficient. 0W: baseline before Lm intervention; 4W: 4-week Lm intervention; 8W: 8-week Lm intervention. CRE, creatinine; TMAO, trimethylamine-N-oxide.
