# Supplementary material for: Multi-omics analysis reveals the efficacy of two probiotic strains in managing feline chronic kidney disease through gut microbiome and host metabolome
Source: Front Vet Sci. 2025 Jun 18;12:1590388. doi: 10.3389/fvets.2025.1590388 (PMC12213445; doi:10.3389/fvets.2025.1590388)
Supplement: Supplementary file 3 [file Table_1.docx]

**Supplementary Table S1. Demographic characteristics of study group**

| **Cases No** | | **Breed** | **Age** | **Weight** | **Sex** | **Feed** | **CKD Stage** |
| --- | --- | --- | --- | --- | --- | --- | --- |
| 1 | | Domestic Shorthair | 14 | 5.9 | Male | Regular | 2 |
| 2 | | Domestic Shorthair | 17 | 3.8 | Female | Renal | 3 |
| 3 | | Domestic Shorthair | 16 | 4.7 | Male | Renal | 3 |
| 4 | | Domestic Shorthair | 6 | 4.1 | Female | Urinary | 2 |
| 5 | | Chinchilla | 13 | 3.2 | Male | Renal | 3 |
| 6 | | Domestic Shorthair | 9 | 6.0 | Male | Regular | 2 |
| 7 | | Domestic Shorthair | 4 | 5.3 | Female | Renal | 2 |
| 8 | | Domestic Shorthair | 11 | 3.3 | Female | Renal | 2 |
| 9 | | Exotic Longhair | 4 | 3.1 | Male | Renal | 2 |
| 10 | | Domestic Shorthair | 7 | 6.2 | Male | Renal | 3 |
| 11 | | Domestic Shorthair | 13 | 8.3 | Male | Renal | 2 |
| 12 | | Domestic Shorthair | 15 | 4.0 | Female | Renal | 2 |
| 13 | | Domestic Shorthair | 16 | 5.4 | Male | Renal | 3 |
| 14 | | Domestic Shorthair | 8 | 4.7 | Male | Renal | 3 |
| Summary | | Domestic Shorthair: 12 (86%)  Chinchilla: 1 (7%)  Exotic Longhair: 1 (7%) | 12 (4–17)* | 4.7 (3.1–8.3)* | Male: 8 (57%)  Female: 6 (43%) | Renal: 11 (79%)  Urinary: 1 (7%)  Regular: 2 (14%) | Stage 2: 8 (57%)  Stage 3: 6 (43%) |
| *The results were displayed as median (range). | | | | | | |  |

**Supplementary Table S2. Serum kidney function indicators and gut-derived uremic toxins in cats with CKD before, during, and after Lm intervention**

| Category | Indicators | Before Lm intervention | In Lm intervention | After Lm intervention |
| --- | --- | --- | --- | --- |
|  |  | 0W | 4W | 8W |
| Kidney function indicator | CRE (mg/dL) | 2.94 (2.50–3.37) | 2.81 (2.32–3.31) | 2.78 (2.31–3.25) |
|  | BUN (mg/dL) | 35.57 (27.69–43.45) | 34.36 (27.69–41.03) | 33.29 (26.35–40.22) |
|  | SDMA (μg/dL) | 17.93 (12.82–23.04) | 13.50 (8.97–18.03) | 15.29 (11.27–19.30) |
| Gut-derived uremic toxin | TMAO (ppb) | 930.7 (507.1–1354.0) | 760.8 (469.1–1053.0) | 801.6 (382.7–1221.0) |
|  | PCS (ppb) | 4663.0 (2330.0–6996.0) | 5576.0 (2950.0–8202.0) | 3964.0 (1890.0–6038.0) |
|  | IS (ppb) | 2677.0 (992.9–4362.0) | 2879.0 (1182.0–4576.0) | 2211.0 (639.6–3783.0) |
|  | PS (ppb) | 617.4 (159.5–1075.0) | 1003.0 (−335.5–2341.0) | 416.3 (153.4–679.2) |

Data were presented as mean (95% conﬁdence intervals).

0W: baseline before Lm intervention; 4W: 4-week Lm intervention; 8W: 8-week Lm intervention.

CRE, creatinine; BUN, blood urea nitrogen; SDMA, symmetric dimethylarginine; TMAO, trimethylamine-N-oxide; IS, indoxyl sulfate; PCS, *p*-cresyl sulfate; PS, phenyl sulfate.

**Supplementary Table S3. Serum and urine biochemical parameters of cats with CKD before, between, and after *Lactobacillus* mix (Lm) intervention**

| Indicators | Before Lm intervention | In Lm intervention | After Lm intervention | *P* value | |
| --- | --- | --- | --- | --- | --- |
|  | 0W | 4W | 8W | 0W vs 4W | 0W vs 8W |
| Hemoglobin (g/dL) | 13.44 (12.01–14.88) | 13.52 (12.22–14.82) | 13.29 (11.96–14.62) | 0.75 | 0.78 |
| Hematocrit (%) | 40.47 (35.82–45.12) | 40.59 (36.28–44.89) | 40.11 (36.03–44.19) | 0.86 | 0.90 |
| Calcium (mg/dL) | 10.85 (10.31–11.39) | 10.41 (9.94–10.89) | 10.47 (9.85–11.09) | 0.01 | 0.03 |
| Phosphate (mg/dL) | 4.18 (3.57–4.78) | 4.16 (3.69–4.64) | 4.22 (3.77–4.67) | 0.83 | 0.53 |
| Ca × P | 45.06 (38.68–51.43) | 43.23 (38.44–48.01) | 44.01 (39.22–48.80) | 0.56 | 0.81 |
| UPC | 0.14 (0.06–0.22) | 0.14 (0.05–0.22) | 0.13 (0.05–0.22) | 0.45 | 0.51 |
| USG | 1.018 (1.010–1.025) | 1.017 (1.013–1.022) | 1.017 (1.013–1.022) | 0.35 | 0.50 |

Data were presented as mean (95% conﬁdence intervals). Variables were tested by the matched-paired Wilcoxon signed-rank test.

0W: baseline before Lm intervention; 4W: 4-week Lm intervention; 8W: 8-week Lm intervention.

Ca × P, calcium–phosphate product; UPC, urine protein creatinine ratio; USG, urine specific gravity.

**Supplementary Table S4. Bacterial species identified in cats with CKD before and after *Lactobacillus* mix (Lm) intervention**

| Relative abundance (%) | Baseline | | Lm intervention | | *P* value |
| --- | --- | --- | --- | --- | --- |
|  | Mean | SD | Mean | SD |  |
| *Adlercreutzia equolifaciens* | 0.0036 | 0.0136 | 0.0045 | 0.0169 | > 0.99 |
| *Agathobaculum desmolans* | 0.0445 | 0.0816 | 0.0330 | 0.0702 | 0.81 |
| *Akkermansia muciniphila* | 0.0024 | 0.0090 | 0.0000 | 0.0000 | > 0.99 |
| *Alistipes putredinis* | 0.0040 | 0.0149 | 0.0028 | 0.0103 | > 0.99 |
| *Allisonella histaminiformans* | 0.0122 | 0.0357 | 0.0160 | 0.0416 | > 0.99 |
| *Allobaculum stercoricanis* | 0.0058 | 0.0217 | 0.0077 | 0.0289 | > 0.99 |
| *Amedibacillus dolichus* | 0.2076 | 0.5797 | 0.0491 | 0.1052 | 0.38 |
| *Aminipila butyrica* | 0.0320 | 0.0924 | 0.0187 | 0.0544 | > 0.99 |
| *Anaerobacterium chartisolvens* | 0.0393 | 0.0797 | 0.0811 | 0.1777 | 0.44 |
| *Anaerobutyricum hallii* | 0.3170 | 0.8207 | 0.2635 | 0.5794 | 0.75 |
| *Anaerofustis stercorihominis* | 0.0101 | 0.0296 | 0.0077 | 0.0235 | 0.50 |
| *Anaeromassilibacillus senegalensis* | 0.0450 | 0.1325 | 0.0091 | 0.0253 | 0.63 |
| *Anaerostipes caccae* | 0.1109 | 0.2852 | 0.1056 | 0.2134 | 0.38 |
| *Anaerostipes hadrus* | 0.0325 | 0.0603 | 0.0284 | 0.0612 | 0.88 |
| ***Anaerotaenia torta*** | **0.2284** | 0.5769 | **0.3330** | 0.7051 | **0.07** |
| *Anaerotignum aminivorans* | 0.0071 | 0.0176 | 0.0060 | 0.0225 | > 0.99 |
| *Anaerotignum lactatifermentans* | 0.0232 | 0.0744 | 0.0323 | 0.0565 | 0.81 |
| *Asaccharobacter celatus* | 0.0013 | 0.0050 | 0.0000 | 0.0000 | > 0.99 |
| *Bacteroides coprocola* | 0.0046 | 0.0124 | 0.0124 | 0.0465 | > 0.99 |
| *Bacteroides faecis* | 0.0000 | 0.0000 | 0.0027 | 0.0100 | > 0.99 |
| *Bacteroides fragilis* | 0.0060 | 0.0223 | 0.0015 | 0.0056 | > 0.99 |
| *Bacteroides ovatus* | 0.0000 | 0.0000 | 0.0132 | 0.0492 | > 0.99 |
| *Bacteroides plebeius* | 0.0061 | 0.0136 | 0.0000 | 0.0000 | 0.25 |
| *Bacteroides stercoris* | 0.0047 | 0.0121 | 0.0087 | 0.0185 | 0.63 |
| *Bacteroides uniformis* | 0.0041 | 0.0109 | 0.0110 | 0.0283 | 0.50 |
| *Bacteroides vulgatus* | 0.0223 | 0.0449 | 0.0382 | 0.0761 | 0.44 |
| *Bacteroides xylanisolvens* | 0.0000 | 0.0000 | 0.0033 | 0.0123 | > 0.99 |
| *Bacteroides xylanolyticus* | 0.0880 | 0.3294 | 0.0182 | 0.0683 | > 0.99 |
| *Barnesiella intestinihominis* | 0.0000 | 0.0000 | 0.0013 | 0.0048 | > 0.99 |
| *Bifidobacterium animalis* | 2.1740 | 8.1043 | 3.2876 | 12.2172 | 0.31 |
| *Bifidobacterium breve* | 0.0258 | 0.0965 | 0.1407 | 0.4150 | 0.50 |
| *Bifidobacterium faecale* | 2.3035 | 8.6097 | 0.4382 | 1.6394 | 0.50 |
| *Bifidobacterium longum* | 0.0960 | 0.3466 | 0.3017 | 1.0457 | 0.25 |
| *Bifidobacterium pseudocatenulatum* | 1.1494 | 4.3007 | 3.9454 | 14.7622 | > 0.99 |
| *Bifidobacterium saeculare* | 4.2548 | 9.8138 | 3.6161 | 7.5878 | 0.57 |
| *Blautia caecimuris* | 10.3365 | 15.0127 | 7.8749 | 9.6981 | 0.58 |
| *Blautia coccoides* | 4.2192 | 5.4350 | 3.3314 | 3.5317 | 0.22 |
| *Blautia glucerasea* | 7.2922 | 13.3720 | 6.6211 | 12.7280 | 0.33 |
| *Blautia hansenii* | 6.5100 | 6.3944 | 7.0070 | 8.0293 | > 0.99 |
| *Blautia hominis* | 0.6837 | 0.8393 | 0.7452 | 1.0072 | 0.91 |
| *Blautia hydrogenotrophica* | 0.6721 | 1.6572 | 0.7097 | 1.3709 | > 0.99 |
| *Blautia luti* | 0.0000 | 0.0000 | 0.2335 | 0.8737 | > 0.99 |
| *Blautia producta* | 0.4065 | 0.8394 | 0.5550 | 1.1263 | 0.38 |
| *Blautia schinkii* | 4.0389 | 4.6558 | 4.3644 | 3.5407 | 0.71 |
| *Blautia wexlerae* | 0.6414 | 1.6145 | 1.6293 | 5.2912 | 0.88 |
| *Butyricicoccus faecihominis* | 0.0083 | 0.0217 | 0.0033 | 0.0124 | 0.75 |
| *Butyricicoccus pullicaecorum* | 0.0021 | 0.0080 | 0.0019 | 0.0071 | > 0.99 |
| *Caecibacter massiliensis* | 0.0000 | 0.0000 | 0.0237 | 0.0886 | > 0.99 |
| *Caecibacterium sporoformans* | 0.0238 | 0.0622 | 0.0056 | 0.0211 | 0.50 |
| *Caproiciproducens galactitolivorans* | 0.0079 | 0.0204 | 0.0000 | 0.0000 | 0.50 |
| *Catenibacterium mitsuokai* | 0.8872 | 2.1098 | 2.5372 | 4.3749 | 0.13 |
| *Christensenella massiliensis* | 0.0000 | 0.0000 | 0.0050 | 0.0137 | 0.50 |
| *Clostridioides difficile* | 0.0021 | 0.0079 | 0.0000 | 0.0000 | > 0.99 |
| *Clostridium baratii* | 0.0348 | 0.1013 | 0.0523 | 0.1536 | 0.50 |
| *Clostridium colicanis* | 0.2788 | 1.0431 | 0.0883 | 0.3042 | > 0.99 |
| *Clostridium dakarense* | 0.0931 | 0.2691 | 0.0000 | 0.0000 | 0.25 |
| *Clostridium disporicum* | 0.1278 | 0.3982 | 0.0744 | 0.1760 | 0.75 |
| *Clostridium innocuum* | 0.1145 | 0.3811 | 0.0686 | 0.2177 | 0.4375 |
| *Clostridium leptum* | 0.4558 | 1.5328 | 0.0939 | 0.2202 | > 0.99 |
| *Clostridium merdae* | 0.0227 | 0.0469 | 0.0127 | 0.0242 | 0.25 |
| *Clostridium methylpentosum* | 0.0034 | 0.0129 | 0.0000 | 0.0000 | > 0.99 |
| *Clostridium paraputrificum* | 0.0000 | 0.0000 | 0.0055 | 0.0207 | > 0.99 |
| *Clostridium perfringens* | 0.8123 | 1.3855 | 0.9316 | 1.7001 | 0.82 |
| *Clostridium sardiniense* | 0.0398 | 0.1489 | 0.0000 | 0.0000 | > 0.99 |
| *Clostridium scindens* | 0.4184 | 0.9777 | 0.0716 | 0.1412 | 0.13 |
| ***Clostridium spiroforme*** | **0.0424** | 0.0733 | **0.0846** | 0.1260 | **0.08** |
| *Clostridium viride* | 0.0772 | 0.2064 | 0.0552 | 0.1607 | 0.75 |
| *Collinsella aerofaciens* | 4.1898 | 7.7719 | 4.8765 | 9.4574 | 0.38 |
| *Collinsella bouchesdurhonensis* | 0.0031 | 0.0114 | 0.0063 | 0.0234 | > 0.99 |
| *Collinsella intestinalis* | 4.6398 | 3.2952 | 4.1593 | 3.3695 | 0.38 |
| *Collinsella massiliensis* | 0.0106 | 0.0398 | 0.0091 | 0.0339 | > 0.99 |
| *Collinsella phocaeensis* | 0.1979 | 0.3666 | 0.2236 | 0.5318 | 0.84 |
| *Collinsella stercoris* | 0.9755 | 2.5872 | 0.9615 | 2.3279 | 0.58 |
| *Collinsella tanakaei* | 5.0878 | 6.9030 | 5.3386 | 6.8487 | 0.83 |
| *Coprococcus comes* | 0.2871 | 0.3270 | 0.5956 | 0.8544 | 0.23 |
| *Coprococcus eutactus* | 0.0015 | 0.0057 | 0.0000 | 0.0000 | > 0.99 |
| *Cuneatibacter caecimuris* | 0.0286 | 0.1018 | 0.0126 | 0.0424 | 0.50 |
| *Cutibacterium acnes* | 0.0000 | 0.0000 | 0.0039 | 0.0145 | > 0.99 |
| *Desulfovibrio piger* | 0.0100 | 0.0254 | 0.0000 | 0.0000 | 0.50 |
| *Dialister invisus* | 0.0465 | 0.1739 | 0.4923 | 1.8421 | > 0.99 |
| *Dorea formicigenerans* | 0.0014 | 0.0051 | 0.0060 | 0.0226 | > 0.99 |
| ***Drancourtella massiliensis*** | **0.4570** | 1.0375 | **1.5567** | 2.5764 | **0.06** |
| *Eggerthella lenta* | 0.1917 | 0.6228 | 0.1670 | 0.5723 | 0.38 |
| *Eisenbergiella massiliensis* | 0.1449 | 0.2456 | 0.0997 | 0.1700 | 0.47 |
| *Emergencia timonensis* | 0.0000 | 0.0000 | 0.0041 | 0.0155 | > 0.99 |
| *Enterocloster bolteae* | 0.0200 | 0.0516 | 0.0071 | 0.0265 | 0.50 |
| *Enterococcus avium* | 0.0414 | 0.1139 | 0.0527 | 0.1446 | 0.50 |
| *Enterococcus cecorum* | 0.0000 | 0.0000 | 0.2330 | 0.7850 | 0.50 |
| *Enterococcus faecalis* | 0.0135 | 0.0242 | 0.2957 | 1.0269 | 0.44 |
| *Enterococcus faecium* | 0.0126 | 0.0389 | 0.0181 | 0.0678 | > 0.99 |
| *Enterococcus hirae* | 0.0084 | 0.0315 | 0.0000 | 0.0000 | > 0.99 |
| *Enterorhabdus caecimuris* | 0.0023 | 0.0086 | 0.0074 | 0.0207 | > 0.99 |
| *Erysipelatoclostridium ramosum* | 0.0574 | 0.1041 | 0.1264 | 0.3196 | 0.69 |
| *Escherichia fergusonii* | 0.0230 | 0.0859 | 0.0000 | 0.0000 | > 0.99 |
| *Eubacterium brachy* | 0.4872 | 1.5081 | 0.2727 | 0.7783 | 0.38 |
| *Eubacterium callanderi* | 1.0004 | 2.9908 | 0.7480 | 2.1376 | 0.47 |
| *Eubacterium coprostanoligenes* | 0.1369 | 0.3644 | 0.0693 | 0.1610 | 0.50 |
| *Eubacterium limosum* | 0.0000 | 0.0000 | 0.0726 | 0.2715 | > 0.99 |
| *Eubacterium tenue* | 0.1104 | 0.4132 | 0.0181 | 0.0679 | > 0.99 |
| *Faecalibacterium prausnitzii* | 0.1043 | 0.2253 | 0.0850 | 0.1636 | > 0.99 |
| *Faecalicatena contorta* | 0.0843 | 0.1415 | 0.1061 | 0.1304 | 0.37 |
| *Faecalicatena fissicatena* | 0.0834 | 0.1789 | 0.1576 | 0.3605 | 0.44 |
| *Faecalicatena orotica* | 0.0038 | 0.0143 | 0.0000 | 0.0000 | > 0.99 |
| *Faecalicoccus pleomorphus* | 0.0409 | 0.1052 | 0.0564 | 0.0814 | 0.47 |
| *Faecalimonas umbilicata* | 1.9691 | 2.3118 | 2.5195 | 4.4087 | 0.95 |
| *Faecalitalea cylindroides* | 0.0078 | 0.0290 | 0.0325 | 0.1215 | > 0.99 |
| *Flavonifractor plautii* | 0.1361 | 0.2579 | 0.1035 | 0.1667 | 0.70 |
| *Fusobacterium varium* | 0.0093 | 0.0349 | 0.0000 | 0.0000 | > 0.99 |
| *Gordonibacter pamelaeae* | 0.0000 | 0.0000 | 0.0091 | 0.0341 | > 0.99 |
| *Gordonibacter urolithinfaciens* | 0.0312 | 0.0619 | 0.0411 | 0.0715 | 0.94 |
| *Helicobacter canis* | 0.0000 | 0.0000 | 0.0019 | 0.0072 | > 0.99 |
| *Holdemanella biformis* | 0.1105 | 0.2834 | 0.4871 | 1.5279 | 0.50 |
| *Holdemania massiliensis* | 0.0000 | 0.0000 | 0.0023 | 0.0085 | > 0.99 |
| *Hungateiclostridium alkalicellulosi* | 0.1356 | 0.5073 | 0.5276 | 1.9742 | > 0.99 |
| *Hungateiclostridium straminisolvens* | 0.0960 | 0.3132 | 0.0995 | 0.3322 | > 0.99 |
| *Ihubacter massiliensis* | 0.0058 | 0.0147 | 0.0098 | 0.0311 | > 0.99 |
| *Intestinimonas butyriciproducens* | 0.0137 | 0.0363 | 0.0046 | 0.0124 | 0.63 |
| *Jeotgalicoccus halotolerans* | 0.0000 | 0.0000 | 0.0030 | 0.0113 | > 0.99 |
| *Lachnoclostridium pacaense* | 0.0842 | 0.2144 | 0.0568 | 0.1190 | 0.75 |
| *Lachnospira eligens* | 0.0027 | 0.0100 | 0.0000 | 0.0000 | > 0.99 |
| *Lacrimispora aerotolerans* | 0.1298 | 0.4493 | 0.0467 | 0.0897 | > 0.99 |
| *Lacrimispora amygdalina* | 0.0645 | 0.1238 | 0.1255 | 0.3115 | 0.81 |
| *Lacrimispora saccharolytica* | 0.3472 | 0.6263 | 0.4147 | 0.6422 | > 0.99 |
| *Lacrimispora xylanolytica* | 0.0000 | 0.0000 | 0.0053 | 0.0197 | > 0.99 |
| *Lactobacillus acidophilus* | 0.0000 | 0.0000 | 0.0101 | 0.0304 | 0.50 |
| *Lentilactobacillus buchneri* | 0.0000 | 0.0000 | 0.0551 | 0.2061 | > 0.99 |
| *Limosilactobacillus coleohominis* | 0.7024 | 2.6283 | 0.0801 | 0.2996 | > 0.99 |
| *Lactobacillus gasseri* | 4.8526 | 18.1569 | 0.8573 | 3.2077 | > 0.99 |
| *Lactobacillus johnsonii* | 3.1454 | 11.7578 | 0.2173 | 0.8130 | 0.25 |
| ***Lacticaseibacillus paracasei*** | **0.0000** | 0.0000 | **0.9162** | 1.5296 | **0.0010** |
| ***Lactiplantibacillus plantarum*** | **0.0000** | 0.0000 | **1.2585** | 1.6991 | **0.0005** |
| *Lactobacillus rogosae* | 0.0000 | 0.0000 | 0.0032 | 0.0121 | > 0.99 |
| *Lacticaseibacillus zeae* | 0.0069 | 0.0257 | 0.0241 | 0.0904 | > 0.99 |
| *Lactococcus lactis* | 0.0000 | 0.0000 | 0.0014 | 0.0052 | > 0.99 |
| *Lactonifactor longoviformis* | 0.0275 | 0.0888 | 0.0028 | 0.0103 | 0.50 |
| *Libanicoccus massiliensis* | 0.0581 | 0.1958 | 0.0359 | 0.1123 | > 0.99 |
| *Longibaculum muris* | 0.0000 | 0.0000 | 0.0974 | 0.3644 | > 0.99 |
| *Macrococcus caseolyticus* | 0.0000 | 0.0000 | 0.0068 | 0.0254 | > 0.99 |
| *Massilimicrobiota timonensis* | 0.0574 | 0.2148 | 0.1227 | 0.4592 | > 0.99 |
| *Mediterraneibacter glycyrrhizinilyticus* | 0.4736 | 0.5785 | 0.5085 | 0.5329 | 0.95 |
| *Megamonas rupellensis* | 0.9704 | 2.4792 | 0.4068 | 1.3464 | 0.50 |
| *Megasphaera elsdenii* | 0.0705 | 0.1737 | 0.0525 | 0.1341 | 0.25 |
| *Megasphaera indica* | 0.0352 | 0.0909 | 0.0229 | 0.0591 | 0.50 |
| *Merdimonas faecis* | 0.2067 | 0.7733 | 0.6931 | 2.5603 | > 0.99 |
| *Monoglobus pectinilyticus* | 0.0193 | 0.0722 | 0.0000 | 0.0000 | > 0.99 |
| *Negativibacillus massiliensis* | 0.1588 | 0.3822 | 0.1061 | 0.1965 | 0.84 |
| *Neglecta timonensis* | 0.0036 | 0.0092 | 0.0053 | 0.0139 | 0.75 |
| *Olsenella profusa* | 0.0185 | 0.0607 | 0.0104 | 0.0388 | 0.50 |
| *Oscillibacter valericigenes* | 0.0175 | 0.0400 | 0.0089 | 0.0335 | 0.75 |
| *Paeniclostridium sordellii* | 0.2564 | 0.9595 | 0.0000 | 0.0000 | > 0.99 |
| *Paludicola psychrotolerans* | 0.0000 | 0.0000 | 0.0026 | 0.0096 | > 0.99 |
| *Parabacteroides distasonis* | 0.0046 | 0.0173 | 0.0015 | 0.0057 | > 0.99 |
| *Parabacteroides merdae* | 0.0106 | 0.0280 | 0.0054 | 0.0155 | 0.88 |
| *Paraprevotella clara* | 0.0045 | 0.0170 | 0.0000 | 0.0000 | > 0.99 |
| *Paraprevotella xylaniphila* | 0.0020 | 0.0075 | 0.0026 | 0.0096 | > 0.99 |
| *Parvibacter caecicola* | 0.0070 | 0.0188 | 0.0045 | 0.0170 | > 0.99 |
| *Pediococcus acidilactici* | 0.2653 | 0.7008 | 0.3567 | 1.0461 | 0.75 |
| *Peptacetobacter hiranonis* | 8.9246 | 14.4037 | 9.1783 | 13.7593 | 0.74 |
| *Peptococcus niger* | 0.9460 | 2.4525 | 0.5916 | 1.1780 | > 0.99 |
| *Peptostreptococcus russellii* | 0.0585 | 0.1137 | 0.0958 | 0.1534 | 0.19 |
| *Phascolarctobacterium faecium* | 0.3482 | 0.9446 | 0.1907 | 0.4226 | 0.56 |
| *Phocea massiliensis* | 0.0159 | 0.0252 | 0.0032 | 0.0121 | 0.13 |
| *Prevotella copri* | 0.2070 | 0.4983 | 0.0643 | 0.2028 | 0.13 |
| *Prevotellamassilia timonensis* | 0.0013 | 0.0050 | 0.0075 | 0.0200 | 0.50 |
| *Pseudopropionibacterium propionicum* | 0.0023 | 0.0086 | 0.0000 | 0.0000 | > 0.99 |
| *Raoultibacter massiliensis* | 0.0021 | 0.0079 | 0.0000 | 0.0000 | > 0.99 |
| *Raoultibacter timonensis* | 0.0013 | 0.0050 | 0.0075 | 0.0243 | > 0.99 |
| *Romboutsia ilealis* | 0.2906 | 0.5251 | 0.3129 | 0.3780 | 0.63 |
| *Romboutsia timonensis* | 0.0000 | 0.0000 | 1.2819 | 4.6677 | 0.50 |
| *Roseburia hominis* | 0.0029 | 0.0109 | 0.0000 | 0.0000 | > 0.99 |
| *Roseburia intestinalis* | 0.0011 | 0.0043 | 0.0000 | 0.0000 | > 0.99 |
| *Rothia nasimurium* | 0.0038 | 0.0143 | 0.0000 | 0.0000 | > 0.99 |
| *Ruminococcus bromii* | 0.0056 | 0.0210 | 0.0000 | 0.0000 | > 0.99 |
| *Ruminococcus callidus* | 0.0015 | 0.0057 | 0.0000 | 0.0000 | > 0.99 |
| ***Ruminococcus gauvreauii*** | **1.4716** | 2.9681 | **1.9519** | 2.8652 | **0.08** |
| *Ruminococcus gnavus* | 2.5202 | 3.6586 | 3.1326 | 4.9283 | 0.36 |
| *Ruminococcus torques* | 0.0432 | 0.0719 | 0.0582 | 0.0881 | 0.58 |
| *Ruthenibacterium lactatiformans* | 0.0763 | 0.1894 | 0.0321 | 0.0667 | 0.30 |
| *Salinicoccus halodurans* | 0.0014 | 0.0051 | 0.0000 | 0.0000 | > 0.99 |
| *Shigella dysenteriae* | 0.0237 | 0.0888 | 0.0000 | 0.0000 | > 0.99 |
| ***Escherichia/Shigella* spp.** | **0.1730** | 0.2570 | **0.0492** | 0.1068 | **0.03** |
| *Shigella sonnei* | 0.0459 | 0.1271 | 0.0075 | 0.0282 | 0.50 |
| ***Slackia faecicanis*** | **0.0214** | 0.0409 | **0.0483** | 0.0650 | **0.06** |
| *Slackia piriformis* | 0.1269 | 0.1657 | 0.1087 | 0.1591 | 0.38 |
| *Snodgrassella alvi* | 0.0000 | 0.0000 | 0.0021 | 0.0078 | > 0.99 |
| *Solobacterium moorei* | 0.0417 | 0.1560 | 0.0711 | 0.2661 | > 0.99 |
| *Spiroplasma chinense* | 0.0048 | 0.0180 | 0.0000 | 0.0000 | > 0.99 |
| *Staphylococcus aureus* | 0.0000 | 0.0000 | 0.0014 | 0.0052 | > 0.99 |
| *Staphylococcus lentus* | 0.0046 | 0.0172 | 0.0000 | 0.0000 | > 0.99 |
| *Streptococcus fryi* | 0.0000 | 0.0000 | 0.0027 | 0.0100 | > 0.99 |
| *Streptococcus minor* | 0.0169 | 0.0634 | 0.0000 | 0.0000 | > 0.99 |
| *Subdoligranulum variabile* | 0.8683 | 1.2019 | 1.4158 | 2.6883 | 0.55 |
| *Sutterella massiliensis* | 0.0000 | 0.0000 | 0.0067 | 0.0250 | > 0.99 |
| *Terrisporobacter mayombei* | 0.0287 | 0.1075 | 0.0072 | 0.0271 | > 0.99 |
| *Turicibacter sanguinis* | 1.1673 | 3.7701 | 0.2038 | 0.5084 | > 0.99 |
| *Tyzzerella nexilis* | 0.0100 | 0.0374 | 0.0026 | 0.0096 | >0.99 |

Variables were tested by the matched-paired Wilcoxon signed-rank test.

**Supplementary Table S5. Selected microbial biomarkers,** **gut microbial functions, and serum metabolites in high responder (HR) and moderate responder (MR) after *Lactobacillus* mix (Lm) intervention**

| **Indicators** | High responder (HR) | | Moderate responder (MR) | | *P* value |
| --- | --- | --- | --- | --- | --- |
|  | Mean | SD | Mean | SD |  |
| **Microbial biomarker**  (Relative abundance %) |  |  |  |  |  |
| *Anaerotaenia torta* | 0.3381 | 0.6690 | 0.0873 | 0.0720 | 0.8674 |
| *Clostridium spiroforme* | 0.1002 | 0.1906 | 0.0776 | 0.0906 | 0.7664 |
| *Drancourtella massiliensis* | 1.8173 | 3.2866 | 1.8153 | 2.4977 | >0.9999 |
| *Ruminococcus gauvreauii* | 2.4980 | 4.2457 | 1.4332 | 1.7935 | 0.8763 |
| *Slackia faecicanis* | 0.0476 | 0.0779 | 0.0466 | 0.0635 | >0.9999 |
| *Lacticaseibacillus paracasei* | 0.9200 | 1.5163 | 0.9011 | 1.7968 | 0.7172 |
| *Lactiplantibacillus plantarum* | 1.8024 | 1.8085 | 1.0746 | 1.8920 | 0.1907 |
| *Escherichia/Shigella* spp. | 0.0758 | 0.1695 | 0.0199 | 0.0527 | 0.7348 |
| **Gut microbial function**  (Relative abundance %) |  |  |  |  |  |
| Phenylalanine metabolism | 0.2122 | 0.0506 | 0.2226 | 0.0447 | 0.8763 |
| Valine, leucine and isoleucine degradation | 0.2660 | 0.0656 | 0.3071 | 0.0550 | 0.6389 |
| Tyrosine metabolism | 0.3016 | 0.0874 | 0.2856 | 0.0576 | 0.6389 |
| Cysteine and methionine metabolism | 0.8774 | 0.0325 | 0.9842 | 0.2849 | >0.9999 |
| Ascorbate and aldarate metabolism | 0.0889 | 0.0371 | 0.0945 | 0.0347 | 0.8763 |
| Citrate cycle (TCA cycle) | 0.4362 | 0.0428 | 0.4339 | 0.1186 | 0.3434 |
| Butanoate metabolism | 0.4061 | 0.0449 | 0.4747 | 0.1284 | 0.4318 |
| Pentose phosphate pathway | 0.7142 | 0.0745 | 0.7024 | 0.0851 | >0.9999 |
| Propanoate metabolism | 0.7110 | 0.1000 | 0.7237 | 0.0603 | 0.6389 |
| Pyruvate metabolism | 1.2016 | 0.1236 | 1.0867 | 0.1816 | 0.4318 |
| Linoleic acid metabolism | 0.0002 | 0.0004 | 5.1612E-5 | 5.3904E-5 | 0.7172 |
| Arachidonic acid metabolism | 0.0039 | 0.0031 | 0.0086 | 0.0104 | 0.4318 |
| Biosynthesis of unsaturated fatty acids | 0.1386 | 0.0358 | 0.1282 | 0.0269 | 0.3434 |
| Fatty acid degradation | 0.2597 | 0.0753 | 0.2807 | 0.0557 | >0.9999 |
| Glycerophospholipid metabolism | 0.4225 | 0.1213 | 0.4594 | 0.0687 | 0.4318 |
| Fatty acid biosynthesis | 0.5275 | 0.0859 | 0.4877 | 0.0457 | 0.2677 |
| **Serum metabolites**  (Log_10_ area intensity) |  |  |  |  |  |
| Capric acid | 4.8904 | 0.4101 | 4.4385 | 0.2259 | 0.0480 |
| L-Tyrosine | 4.8985 | 0.0982 | 4.8450 | 0.1584 | 0.5303 |
| L-Arginine | 4.8170 | 0.0824 | 4.8180 | 0.3131 | >0.9999 |
| Glycine | 5.2413 | 0.0850 | 5.1442 | 0.3857 | >0.9999 |
| cis-Aconitic acid | 5.3549 | 0.1026 | 5.5127 | 0.3735 | 0.4318 |
| DL-Glutamate | 5.2862 | 0.0672 | 5.3807 | 0.2532 | 0.2020 |
| Dihydrobiopterin | 4.0698 | 0.5116 | 4.2233 | 0.4304 | 0.8763 |
| N-Methylhydantoin | 4.2981 | 0.1426 | 4.2011 | 1.1386 | 0.4318 |
| Isocitric acid | 5.5645 | 0.4032 | 5.8165 | 0.1766 | 0.2677 |
| 3-Methoxytyramine | 4.5463 | 0.1088 | 4.7100 | 0.4432 | 0.2020 |
| Acetylcholine | 4.4906 | 0.0939 | 4.6412 | 0.4069 | 0.4318 |
| L-Valine | 3.6941 | 0.2270 | 3.8198 | 0.2258 | 0.2677 |

Variables were tested by the Wilcoxon rank-sum test.

**Supplementary Table S6. Bacterial families identified in cats with CKD before and after *Lactobacillus* mix (Lm) intervention**

| Relative abundance (%) | Baseline | Lm intervention | *P* value |
| --- | --- | --- | --- |
|  | Mean | Mean |  |
| *Lachnospiraceae* | 39.9 | 39.2 | 0.56 |
| *Coriobacteriaceae* | 15.1 | 15.6 | > 0.99 |
| *Bifidobacteriaceae* | 10.0 | 11.7 | 0.25 |
| *Peptostreptococcaceae* | 9.6 | 10.9 | 0.79 |
| *Lactobacillaceae* | 9.0 | 3.8 | > 0.99 |
| *Ruminococcaceae* | 3.4 | 5.3 | 0.89 |
| *Erysipelotrichaceae* | 2.6 | 3.8 | 0.50 |
| *Oscillospiraceae* | 2.6 | 3.2 | 0.45 |
| *Clostridiaceae* | 2.1 | 1.5 | 0.84 |
| *Eubacteriaceae* | 1.7 | 1.2 | 0.50 |
| *Peptococcaceae* | 0.95 | 0.59 | 0.45 |
| *Selenomonadaceae* | 0.97 | 0.41 | 0.03 |
| *Hungateiclostridiaceae* | 0.27 | 0.71 | 0.16 |
| *Eggerthellaceae* | 0.38 | 0.39 | 0.13 |
| *Veillonellaceae* | 0.16 | 0.61 | 0.17 |
| *Enterococcaceae* | 0.08 | 0.60 | 0.88 |
| *Acidaminococcaceae* | 0.35 | 0.19 | > 0.99 |
| *Enterobacteriaceae* | 0.27 | 0.06 | > 0.99 |
| *Prevotellaceae* | 0.21 | 0.07 | 0.22 |
| *Bacteroidaceae* | 0.14 | 0.11 | 0.86 |
| *Atopobiaceae* | 0.08 | 0.05 | 0.19 |
| *Eubacteriales, not assigned to family* | 0.03 | 0.02 | > 0.99 |
| *Tannerellaceae* | 0.02 | 0.01 | > 0.99 |
| *Streptococcaceae* | 0.02 | 0.00 | > 0.99 |
| *Staphylococcaceae* | 0.01 | 0.01 | 0.39 |
| *Desulfovibrionaceae* | 0.01 | 0.00 | 0.24 |
| *Fusobacteriaceae* | 0.01 | 0.00 | 0.13 |
| *Rikenellaceae* | 0.00 | 0.00 | > 0.99 |
| *Sutterellaceae* | 0.00 | 0.01 | > 0.99 |
| *Propionibacteriaceae* | 0.00 | 0.00 | 0.02 |
| *Christensenellaceae* | 0.00 | 0.00 | 0.50 |
| *Spiroplasmataceae* | 0.00 | 0.00 | > 0.99 |
| *Micrococcaceae* | 0.00 | 0.00 | 0.88 |
| *Akkermansiaceae* | 0.00 | 0.00 | > 0.99 |
| *Neisseriaceae* | 0.00 | 0.00 | > 0.99 |
| *Helicobacteraceae* | 0.00 | 0.00 | 0.81 |
| *Barnesiellaceae* | 0.00 | 0.00 | 0.81 |

Variables were tested by the matched-paired Wilcoxon signed-rank test.
